# Two Birds With One Stone: A Novel Nanocomposite Combining Silicon Nanoparticles and Carbon‐Doped Titania as the Fluorescence Probe and Visible Light Photocatalyst for Simultaneous Detection and Removal of Oxytetracycline

**DOI:** 10.1002/advs.202410306

**Published:** 2024-12-09

**Authors:** Liyun Ma, Zhi Li, Yiyi Shi, Yuchen Xie, Ming Jiang, Xu Yu, Li Xu

**Affiliations:** ^1^ Department of Pharmacy Union Hospital Tongji Medical College Huazhong University of Science and Technology Wuhan 430022 P. R. China; ^2^ Tongji School of Pharmacy Huazhong University of Science and Technology Wuhan 430030 P. R. China

**Keywords:** carbon‐doped titania, fluorescence sensor, oxytetracycline, silicon nanoparticles, visible light photocatalyst

## Abstract

The monitoring and removal of environmental pollutants are vital for the sustainable development of society. Herein, a novel nanocomposite combining silicon nanoparticles and carbon‐doped titania, C‐TiO_2_/SiNPs, is designed as a fluorescence probe and visible‐light photocatalyst to simultaneously monitor and degrade oxytetracycline (OTC). C‐TiO_2_/SiNPs emit blue fluorescence, which can be quenched by OTC through inner filter effect. Meanwhile, green fluorescence is turned on upon OTC addition by aggregation‐induced emission. Thus, C‐TiO_2_/SiNPs successfully act as a fluorescence colorimetric probe for quantitation and semi‐quantitation of OTC. Additionally, C‐TiO_2_/SiNPs show adorable visible‐light photocatalytic activity toward OTC, achieving a removal rate of 93.3% in 30 min. This process is driven by the superoxide radicals, holes and hydroxyl radicals produced by C‐TiO_2_/SiNPs under visible light irradiation. The total toxicities obviously reduce after OTC is degraded by C‐TiO_2_/SiNPs. The concentration during the OTC degradation can be estimated by naked eyes through the change of fluorescence color, but also precisely quantified by a fluorometer. This study innovatively develops a one‐stone‐two‐birds strategy to achieve simultaneous degradation and in situ real‐time detection of OTC, paving a promising paradigm for the highly efficient monitoring and removal of residual drugs.

## Graphical abstract

A bifunctional nanocomposite combining silicon nanoparticles and C‐doped titania (C‐TiO_2_/SiNPs) is innovatively designed, whose blue fluorescence can be quenched by oxytetracycline (OTC) with turn‐on green fluorescence. Besides, C‐TiO_2_/SiNPs has visible‐light photocatalytic activity to assist the degradation of OTC. Thus, the degradation and in‐situ real time detection of OTC are simultaneously achieved by C‐TiO_2_/SiNPs.

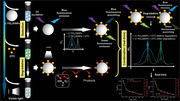

## 1 Introduction

With the advancement of social globalization, pollution from residual pharmaceuticals in environment is challenging around the world.^[^ ^1^ ^]^ As a class of broad‐spectrum antibiotics, tetracyclines (TCs) are extensively used and ubiquitous in the environment, with residual concentrations as high as up to mg L^−1^.^[^ ^2^, ^3^ ^]^ Due to their persistence, the residual TCs exist and transport in the environment, potentially causing negative effects on humans and ecosystems because of their long‐term cumulative effects, such as hepatotoxicity, genotoxicity, microbial resistance issue and so forth.^[^ ^2^, ^3^ ^]^ For instance, the reproductive effects for oxytetracycline (OTC) were observed at the concentration of 5–50 mg L^−1^.^[^ ^4^ ^]^ Therefore, monitoring and removing TCs residues from the environment are of great significance for the sustainability of ecosystems and public health.

Up to now, numerous methods have been established for detecting TCs, such as high‐performance liquid chromatography (HPLC),^[^ ^5^ ^]^ liquid chromatography‐tandem mass spectrometry (LC‐MS/MS),^[^ ^6^ ^]^ and immunoassay,^[^ ^7^ ^]^ etc. Although these methods exhibit high selectivity and sensitivity, they are often time‐consuming, labor‐intensive, costly, and require complicated instruments and professional trained personnel, which especially limits their on‐site applications.^[^ ^8^ ^]^ Therefore, more convenient detection methods, especially for on‐site detection purpose, are still highly desired.

Regarding the removal of TCs, photocatalytic technology has attracted heightened attention in terms of its high efficiency, cost effectiveness, and environmental friendliness.^[^ ^9^ ^]^ Among others, titanium dioxide (TiO_2_) is regarded as one kind of the most often used photocatalysts in light of its prominent photocorrosion stability, non‐toxicity and low cost. However, due to its broad bandgap energy (E_g_) (≈3.1 eV),^[^ ^10^ ^]^ the photocatalytic activity of TiO_2_ is somewhat restricted, as ultraviolet (UV) light is normally necessary for its photocatalytic function. As well known, UV light accounts for only 5% solar radiation, which is far lower than 50% contributed by visible light.^[^ ^11^ ^]^ In order to utilize the energy of the visible light and reduce cost, TiO_2_ with the capability to absorb visible light is in high demand.

Moreover, in the vast majority of cases, the detection and removal of the residual pharmaceuticals are carried out independently.^[^ ^9^, ^12^ ^]^ Actually, these two operations are dependent on each other in practical applications. The environmental governance on the removal of pollutants should be based on the monitoring (detection) results, while detection should evaluate the pollution levels and assess the effectiveness of environmental governance. Therefore, establishing an integrated technology capable of simultaneously detecting and removing residual pharmaceuticals would be a valuable approach, particularly for achieving in situ monitoring during the removal process.

Recently, researchers have increasingly devoted themselves to this endeavor.^[^ ^13^, ^14^ ^]^ For instance, Han et al. used TiO_2_, silver (Ag) and polyimide to prepare a novel polyimide/TiO_2_/Ag organic–inorganic ternary flexible composite microfibers, which highly efficient photocatalytic degrade and sensitively detect tetracycline (TC) by surface‐enhanced Raman spectroscopy.^[^ ^13^ ^]^ In another attempt, a multifunctional composite, CaO_2_‐loaded Cu‐MOF nanosheets, was fabricated to achieve fluorescence detection and Fenton‐like degradation of TC.^[^ ^14^ ^]^ Nevertheless, in these cases, detection and degradation were still treated separately and not truly synchronized, and in situ monitoring of the degradation process in real time was seldom reported and still lacking. Recently, prussian blue bimetallic analog‐modified TiO_2_ particles were successfully prepared for the simultaneous detection and removal of cyanide.^[^ ^15^ ^]^ Also, our group constructed multifunctional sulfur (S) and nitrogen (N) co‐doped silicon nanoparticles (SN‐SiNPs) to simultaneously achieve photo‐degradation of TCs and visually detect their concentrations in real time.^[^ ^16^ ^]^ This silicon‐based nanomaterial was applied not only as a ratiometric fluorescence probe to detect TCs by fluorescence‐quenching the blue fluorescence of SN‐SiNPs via inner filter effect (IFE) and turning on green fluorescence of TCs via aggregation induced emission (AIE), but also as a photocatalyst to degrade TCs. However, SN‐SiNPs only displayed photocatalytic ability under UV light and took long time to achieve a satisfactory removal rate. For example, to obtain the removal rate of ≈80% for OTC, an exposure time of 240 min to UV light was required.

**Scheme 1:**
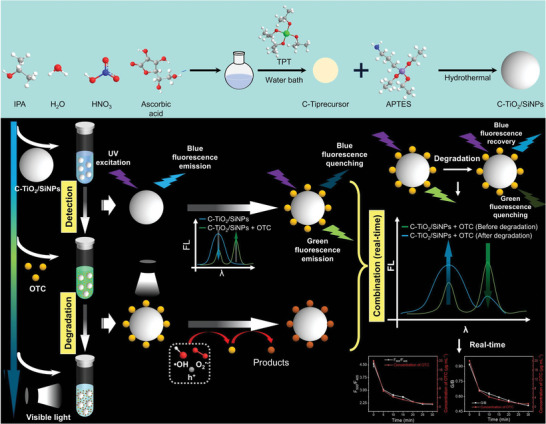
Preparation process and “two‐in‐one” applications of C‐TiO_2_/SiNPs.

As our continuous and innovative endeavor to detect and degrade residual pharmaceuticals for environmental remediation, enhance the catalytic efficiency and utilize the visible light to save the energy, herein, a novel SiNPs and C‐doped TiO_2_ nanocomposite, named as C‐TiO_2_/SiNPs, was elaborately designed, prepared and applied (**Scheme** **1**). Using a one‐pot hydrothermal method, 3‐aminopropyl triethoxysilane (APTES) reacted with C‐doped TiO_2_ (C‐TiO_2_) precursor, generating C‐TiO_2_/SiNPs with blue fluorescence and visible light photocatalytic ability. The detection and photocatalytic performance of C‐TiO_2_/SiNPs was investigated in detail, using OTC as a model analyte. The mechanisms behind the detection and photocatalysis were systematically explored. The degradation pathway and total toxicity assessment after degradation were studied as well. The present work provided a “one‐stone‐two‐birds” strategy for simultaneous in‐situ real‐time visual detection and removal of residual OTC.

## 2 Results and Discussion

### 2.1 Preparation and Characterization of C‐TiO_2_/SiNPs

In order to utilize the energy of visible light, one effective method is to narrow the E_g_ of TiO_2_, which could be realized by forming defects.^[^ ^17^ ^]^ The formation of defects is often achieved through element doping, such as C, S, N or other elements, which mainly replace the oxygen of TiO_2_ to form the oxygen defects.^[^ ^18^, ^19^, ^20^ ^]^ Element doped TiO_2_ generally has a narrower E_g_ and is endowed improved catalytic performance. Thus, herein, the C‐TiO_2_ precursor was deliberately used to generate C‐doped TiO_2_. On the other hand, benefitting from their outstanding optical properties, tunable fluorescence emission, good photo‐stability and large Stokes shift, silicon nanomaterials have been widely used for fluorescence sensing and fluorescence imaging, and related applications.^[^ ^21^, ^22^ ^]^ Some published studies have successfully implemented silicon nanomaterials as fluorescence probes for detecting TCs,^[^ ^16^ ^]^ nitrophenol isomers^[^ ^23^ ^]^ and metals.^[^ ^24^ ^]^ Considering these facts, SiNPs were herein applied in combination with C‐TiO_2_ as a fluorescence probe to detect OTC.

**Figure 1:**
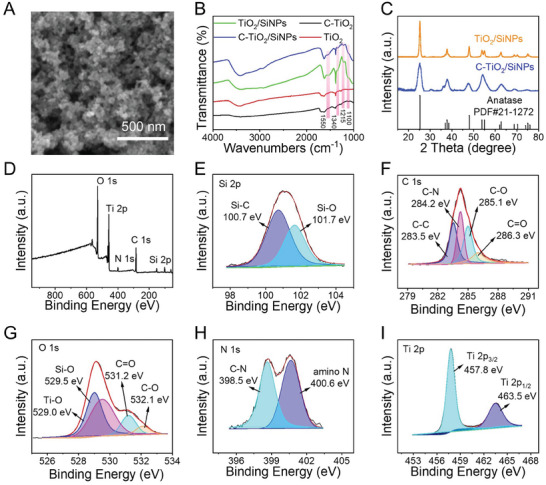
Characterization of C‐TiO_2_/SiNPs. A) The SEM image of C‐TiO_2_/SiNPs. B) FT‐IR spectra of various materials as noted. C) The XRD spectra of C‐TiO_2_/SiNPs and TiO_2_/SiNPs. Full‐scan D) and narrow‐scan XPS spectra of Si 2p E), C 1s F), O 1s G), N 1s H) and Ti 2p I) for C‐TiO_2_/SiNPs.

C‐TiO_2_/SiNPs appeared aggregated ultra‐small nanoparticles (**Figure** **1**A). Four peaks at 1550 cm^−1^ (‐NH), 1340 cm^−1^ (C─O─C), 1215 cm^−1^ (Ti─O─C) and 1100 cm^−1^ (Si─O─Si) were found in C‐TiO_2_/SiNPs and TiO_2_/SiNPs (Figure 1B), indicating that SiNPs (derived from APTES) were involved in these two materials.^[^ ^10^, ^11^, ^16^, ^25^ ^]^ According to the results of elemental analysis (EA) (Table S1, Supporting Information), the contents of N in C‐TiO_2_/SiNPs (3.32%) and TiO_2_/SiNPs (2.63%) were obviously higher than those in C‐TiO_2_ (0.21%) and TiO_2_ (0.24%), which might be ascribed to the SiNPs originating from APTES containing high content of N. Moreover, as displayed in X‐ray diffraction (XRD) patterns (Figure 1C), a typical anatase phase (PDF#21‐1272) was disclosed for both TiO_2_/SiNPs and C‐TiO_2_/SiNPs, indicating the potential catalytic activity of these two materials due to their high purity of crystalline structure.^[^ ^26^ ^]^ The full survey and narrow scan X‐ray photoelectron spectroscopic (XPS) patterns for C‐TiO_2_/SiNPs are shown in Figure 1D‐I. The full survey spectrum (Figure 1D) disclosed the existence of silicon (Si 2p, 6.02%), carbon (C 1s, 31.44%), oxygen (O 1s, 41.73%), nitrogen (N 1s, 5.51%) and titanium (Ti 2p, 15.30%) in C‐TiO_2_/SiNPs. The deconvoluted XPS spectrum of Si 2p (Figure 1E) showed peaks of Si─C (100.7 eV) and Si─N (101.7 eV).^[^ ^10^, ^25^ ^]^ The broad peak of C 1s (Figure 1F) was deconvoluted into four peaks of C─C (283.5 eV), C─N (284.2 eV), C─O (285.1 eV) and C═O (286.3 eV).^[^ ^11^, ^25^ ^]^ The high‐resolution spectrum of O 1s (Figure 1G) also presented four peaks as Ti─O (529.0 eV), Si─O (529.5 eV), C═O (531.2 eV) and C─O (532.1 eV).^[^ ^25^, ^27^ ^]^ Both the XPS patterns of C 1s and O 1s revealed the existence of C─O and C═O bond in C‐TiO_2_/SiNPs. In the narrow scan spectrum of N 1s (Figure 1H), C─N (398.5 eV) and amino N (400.6 eV) were observed. The Ti 2p spectrum (Figure 1I) was fitted to two peaks at 457.8 and 463.5 eV, corresponding to Ti 2p_3/2_ and Ti 2p_1/2_, respectively.^[^ ^28^ ^]^ Based on all the above characterization results, it was concluded that C‐TiO_2_/SiNPs nanocomposites were successfully prepared.

**Figure 2:**
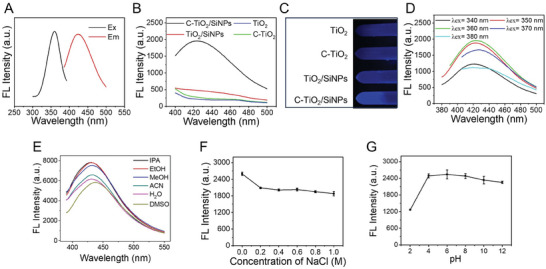
The fluorescence properties of C‐TiO_2_/SiNPs. A) Excitation (E_x_) and emission (E_m_) fluorescence spectra of C‐TiO_2_/SiNPs. B) Comparison of emission spectra of different materials (λ_ex_ = 365 nm). C) Photos of noted materials under UV light (λ = 365 nm) irradiation. The emission spectra of C‐TiO_2_/SiNPs under different excitation wavelengths D) and in different solvents as indicated (λ_ex_ = 365 nm) E). The fluorescence intensity of C‐TiO_2_/SiNPs (λ_ex_ = 365 nm, λ_em_ = 430 nm) in different concentrations of NaCl F) and pHs G).

Moreover, the fluorescence properties of C‐TiO_2_/SiNPs were studied, using TiO_2_/SiNPs, C‐TiO_2_ and TiO_2_ for comparison. As shown in **Figure** **2**A, the maximum excitation (E_x_) and emission (E_m_) wavelengths of C‐TiO_2_/SiNPs were ≈365 nm and ≈430 nm, respectively. Under the excitation of 365 nm, TiO_2_/SiNPs emitted faint blue fluorescence, and C‐TiO_2_ and TiO_2_ emitted bare fluorescence, while the strongest blue fluorescence was observed for C‐TiO_2_/SiNPs (Figure 2B,C). The phenomena illustrated that fluorescence occurred only when APTES was involved in the material preparation. The strengthened fluorescence of C‐TiO_2_/SiNPs compared to TiO_2_/SiNPs might be attributed to the C‐doping involved in C‐TiO_2_/SiNPs. In addition, the fluorescence of C‐TiO_2_/SiNPs was nearly independent of excitation wavelength (Figure 2D). The solvent effect on the fluorescence intensity of C‐TiO_2_/SiNPs was studied in water (H_2_O) and five commonly‐used organic solvents (isopropanol (IPA), ethanol (EtOH), methanol (MeOH), acetonitrile (ACN) and dimethyl sulfoxide (DMSO)). As revealed in Figure 2E, the fluorescence intensity of C‐TiO_2_/SiNPs varied among these studied solvents, but still remained satisfactory fluorescence intensities. The maximum E_m_ wavelengths of C‐TiO_2_/SiNPs remained constant (≈430 nm) in most studied solvents, but underwent a slight redshift in DMSO. Although fluorescence intensity was affected by salinity and pH, it remained relatively stable when the sodium chloride (NaCl) concentration exceeded 0.2 M (Figure 2F) and across pHs of 4–12 (Figure 2G). The relatively stable fluorescence property of C‐TiO_2_/SiNPs guaranteed its applicability for practical use.

### 2.2 Detection of OTC Using C‐TiO_2_/SiNPs as the Probe

**Figure 3:**
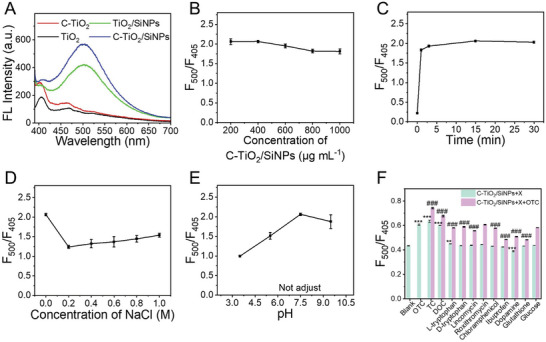
The optimization of detection of OTC using C‐TiO_2_/SiNPs as the probe. A) Fluorescence responses of different materials to OTC. Optimization of the concentration of C‐TiO_2_/SiNPs B), incubation time C), salinity D) and pH E). F) Selectivity of C‐TiO_2_/SiNPs for OTC over other coexisting analytes (X), and the coexisting interference of these studied analytes. (n = 3 independent samples, data are presented as the mean ± S.D., one‐way ANOVA, ** *p*< 0.01, *** or ^###^ *p*< 0.001.).

In our previous study, in light of the AIE effect of TCs themselves, the ratiometric fluorescence probe was constructed based on a single probe for TCs detection.^[^ ^16^ ^]^ As this was a self‐calibration process, it provided reliable results. Herein, C‐TiO_2_/SiNPs were also considered as a ratiometric fluorescence probe for TCs detection. Taking OTC as the model target, both C‐TiO_2_/SiNPs and TiO_2_/SiNPs exerted quenched blue fluorescence (405 nm) and newly turned‐on green fluorescence (500 nm) upon the interaction with OTC, with the former showing stronger green fluorescence; however, this phenomenon was not observed in C‐TiO_2_ or TiO_2_ (**Figure** **3**A). The Stern‐Volmer plot (Text S1, Supporting Information) was then used to tell whether the quenching process was static or dynamic.^[^ ^29^ ^]^ As shown in Figure S1A (Supporting Information), a linearity between the quenching ratio and concentration of OTC (0.078–20 µg mL^−1^) was obtained (R^2^ = 0.9990), indicating that the quenching fluorescence of C‐TiO_2_/SiNPs by OTC was a static quenching process.^[^ ^29^, ^30^ ^]^ Meanwhile, as depicted in Figure S1B (Supporting Information), there was a significant overlap between the UV–vis absorbance spectrum of OTC and the excitation spectrum of C‐TiO_2_/SiNPs, disclosing that the IFE may be responsible for the blue fluorescence quenching of C‐TiO_2_/SiNPs. In addition, the lifetime of C‐TiO_2_/SiNPs in the absence and presence of OTC was almost the same to each other, measured as 4.07 ns and 4.12 ns, respectively (Figure S1C, Supporting Information), implying that no significant existence of electron‐transfer process between C‐TiO_2_/SiNPs and OTC, and also reflecting the fluorescence quenching mechanism of IFE in this detection. More intuitively, as photographed in Figure S1D (Supporting Information), C‐TiO_2_/SiNPs appeared white under visible light and emitted blue fluorescence under UV light (λ = 365 nm). However, when it was mixed with OTC (C‐TiO_2_/SiNPs + OTC), C‐TiO_2_/SiNPs + OTC turned yellow under visible light and emitted green fluorescence under UV light (λ = 365 nm). Combining the fact that OTC is a yellow crystalline powder with a tetraphenylene structure, it is speculated that the green fluorescence of C‐TiO_2_/SiNPs + OTC might result from the adsorption and aggregation of OTC due to interaction (such as hydrogen bonding, etc.) between OTC and the surface functional groups of C‐TiO_2_/SiNPs,^[^ ^31^, ^32^ ^]^ which was regarded as AIE.^[^ ^33^, ^34^ ^]^ This finding agreed well with our previous observation.^[^ ^16^ ^]^ The blue fluorescence of C‐TiO_2_/SiNPs was quenched by OTC through IFE, while the green fluorescence was turned on upon the interaction between OTC and C‐TiO_2_/SiNPs by AIE. Thus, C‐TiO_2_/SiNPs were suitable as an appropriate probe for ratiometric fluorescence detection of OTC.

#### 2.2.1 Optimization of Detection Conditions

To obtain the optimum sensing performance towards OTC, the detection conditions were systematically examined, including the concentration of C‐TiO_2_/SiNPs, incubation time, salinity and pHs.

The effect of the concentration of C‐TiO_2_/SiNPs on detection fluorescence signal was depicted in Figure 3B. Without C‐TiO_2_/SiNPs, no fluorescence was observed. In the presence of C‐TiO_2_/SiNPs, obvious fluorescence appeared, in which the value of F_500_/F_405_ remained almost stable in the case of 200 and 400 µg mL^−1^ of C‐TiO_2_/SiNPs, and then decreased slightly with the increasing C‐TiO_2_/SiNPs. The phenomenon might be explained by the availability of more binding sites with the increasing dosage of C‐TiO_2_/SiNPs initially, which strengthened the response upon the interaction with OTC. However, with the further increasing C‐TiO_2_/SiNPs, the blue fluorescence of C‐TiO_2_/SiNPs itself was intensified, while the green fluorescence induced by OTC remained almost constant; thus, the value of F_500_/F_405_ decreased. Ultimately, 400 µg mL^−1^ was chosen for subsequent experiments.

The effect of incubation time on the detection fluorescence signal was depicted in Figure 3C. The response of C‐TiO_2_/SiNPs to OTC was rapidly enhanced in 10 s, reaching maximum at 15 min and remaining stable for at least 30 min. This might be due to the transient adsorption of OTC to C‐TiO_2_/SiNPs, making this immediate response suitable for rapid detection. To ensure operational convenience and repeatability, 15 min was chosen for the following tests.

Subsequently, the salinity was optimized with NaCl concentration ranging from 0 to 1 M. As shown in Figure 3D, the ratio value of F_500_/F_405_ was the highest without the addition of NaCl. The response was sharply decreased upon the addition of NaCl (from 0 to 0.2 M), and then remained almost constant with further increasing NaCl concentration. The reason behind this change may be related to the response of C‐TiO_2_/SiNPs to NaCl (Figure 2F). Ultimately, no NaCl addition was preferred.

For the effect of pH, the value of F_500_/F_405_ was the highest when the pH of the detection system was not adjusted (herein pH 7.5) (Figure 3E). This phenomenon might be attributed to the ionization states of the functional groups on the surface of C‐TiO_2_/SiNPs and OTC at different pHs. C‐TiO_2_/SiNPs were positively charged at pH<8.5 and negatively charged at pH>8.5 (Figure S2, Supporting Information). As OTC is a triprotic ampholyte compound with the isoelectric point of pH 5.2,^[^ ^4^ ^]^ it was positively charged under investigated pH of 3.5 and negatively charged under the pHs of 5.5, 7.5 and 9.5, respectively. Therefore, the interaction between C‐TiO_2_/SiNPs and OTC might reach the strongest at pH 7.5, leading to the strongest responsive signal.

Overall, based on the above analysis, the optimal detection condition for OTC determination was at pH 7.5, with an incubation time of 15 min and without the addition of NaCl.

Furthermore, the detection selectivity of this C‐TiO_2_/SiNPs probe for OTC was assessed. As shown in Figure 3F, the TCs, including TC, OTC and doxycycline (DOC) triggered significantly higher responses, further demonstrating the detectability of the C‐TiO_2_/SiNPs probe for TCs. Little changes were found for the other investigated substances, suggesting that C‐TiO_2_/SiNPs possessed satisfactory selectivity for the detection of TCs. What's more, the investigated possible coexisting analytes had minor effect on the detectability of C‐TiO_2_/SiNPs towards OTC, even in the case that the concentration of the former ones (50 µg mL^−1^) was ten‐fold of OTC (5 µg mL^−1^).

#### 2.2.2 Evaluating Sensing Performance of the C‐TiO_2_/SiNPs Probe

**Figure 4:**
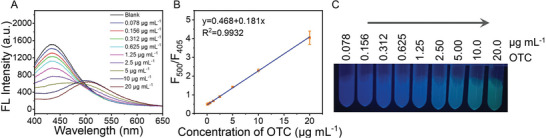
The detection performance of C‐TiO_2_/SiNPs to OTC. A) The fluorescence spectra of C‐TiO_2_/SiNPs in response to OTC. B) The linearity of F_500_/F_405_ value to the concentrations of OTC. C) The photos of C‐TiO_2_/SiNPs with different concentrations of OTC under UV light (λ = 365 nm).

Under the optimized detection conditions, the sensing performance of C‐TiO_2_/SiNPs for OTC determination was evaluated. As the concentration of OTC increased (**Figure** **4**A), the fluorescence intensity at 405 nm was progressively weakened, while the fluorescence at 500 nm was enhanced. A linearity between the F_500_/F_405_ values and concentration of OTC was obtained in the range of 0.078‐20 µg mL^−1^, with a good coefficient of determination (r^2^ = 0.9932) (Figure 4B) and a limit of detection (LOD) of 0.01 µg mL^−1^.

Meanwhile, as shown in Figure 4C, with the elevated concentrations of OTC, the fluorescence color changed from blue to green, and the standard colorimetric card for OTC was thus established. Based on this criterion, C‐TiO_2_/SiNPs could realize the semi‐quantification of OTC by fluorescence color variations for visual detection.

### 2.3 Degradation of OTC Using C‐TiO_2_/SiNPs as the Photocatalyst

#### 2.3.1 Catalytic Performance of C‐TiO_2_/SiNPs

**Figure 5:**
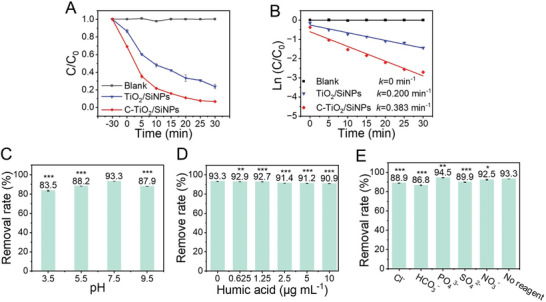
Photocatalytic performance of C‐TiO_2_/SiNPs. A) Photocatalytic degradation curves and B) Pseudo‐first‐order kinetics for the degradation of OTC by different photocatalysts under visible light irradiation. Removal rates towards OTC using C‐TiO_2_/SiNPs as the photocatalyst C) under different pHs, D) in the presence of humic acid of different concentrations, and E) with various anions (10 mM). (n = 3 independent samples, data are presented as the mean ± S.D., one‐way ANOVA, *p < 0.05, **p < 0.01, ***p < 0.001.).

To evaluate the photocatalytic performance of the prepared materials, degradation of OTC was studied in the presence of different catalysts under LED irradiation. As shown in the kinetic curves (**Figure** **5**A), OTC could be degraded by C‐TiO_2_/SiNPs and TiO_2_/SiNPs under visible light irradiation, with removal rates of 93.3% and 76.1% within 30 min, respectively. The pseudo‐first‐order model was utilized to quantitatively evaluate the photocatalytic activity of these two photocatalysts (Figure 5B). The apparent rate constants of C‐TiO_2_/SiNPs and TiO_2_/SiNPs toward OTC were obtained as 0.383 min^−1^ and 0.200 min^−1^, respectively. Obviously, C‐TiO_2_/SiNPs exerted higher photocatalytic activity and removal rate for OTC, better than those of our previous SN‐SiNPs.^[^ ^16^ ^]^ The total organic carbon (TOC) of OTC before and after photocatalytic degradation within 60 min showing a slight downward trend, while the total nitrogen (TN) content remained almost unchanged (Figure S3, Supporting Information), indicating that most of OTC transformed into other forms present in the solution and longer degradation time may be required to make the transformation products be mineralized.^[^ ^35^ ^]^ Additionally, the removal rate of C‐TiO_2_/SiNPs towards OTC remained stable in a wide pH range (Figure 5C). Moreover, the influence of the common substances in real environmental water, e.g. humic acid and some anions, on the removal rates of C‐TiO_2_/SiNPs towards OTC was also investigated. As shown in Figure 5D, humic acid with varied concentrations (0.625 to 10 µg mL^−1^) had little effect on the removal rates of OTC by the C‐TiO_2_/SiNPs/LED system. Although the removal rates were slightly affected by the studied anions of high concentrations (10 mM), the removal rates were still acceptable (Figure 5E). These results illustrated that C‐TiO_2_/SiNPs exhibited potential applicability for removing OTC in real environmental water.

#### 2.3.2 Possible Photocatalytic Mechanism

**Figure 6:**
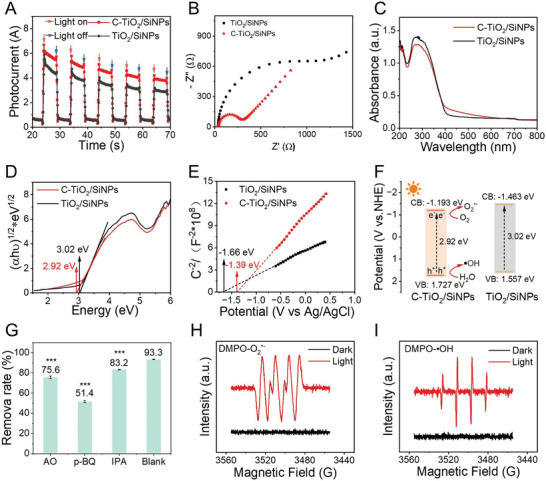
A) Photocurrent responses spectra, B) EIS Nyquist plots, C) UV–vis DRS spectra, D) transferred Tauc plots, E) Mott‐Schottky plots and F) schematic energy‐band diagrams of C‐TiO_2_/SiNPs and TiO_2_/SiNPs. G) The removal rates of OTC by C‐TiO_2_/SiNPs in the presence of different reactive species scavengers under visible‐light irradiation. ESR spectra of H) O_2_^•−^ and I) ⋅OH. (n = 3 independent samples, data are presented as the mean ± S.D., one‐way ANOVA, ***p < 0.001).

To reveal the underlying reasons for the excellent photocatalytic performance of C‐TiO_2_/SiNPs, the optical and photoelectrochemical characterizations were carried out. The transient photocurrent response curves and electrochemical impedance spectra (EIS) of C‐TiO_2_/SiNPs and TiO_2_/SiNPs are shown in **Figure** **6**A,B. Compared with TiO_2_/SiNPs, C‐TiO_2_/SiNPs exhibited stronger photocurrent intensity and smaller impedance, implying easier electronic conduction and separation of photogenerated electron‐hole pairs, which would facilitate photocatalysis. The UV–vis diffuse reflectance spectroscopy (UV–vis DRS) of C‐TiO_2_/SiNPs and TiO_2_/SiNPs are displayed in Figure 6C. The absorption of C‐TiO_2_/SiNPs in the wavelength range of 400–700 nm (visible light) was lightly stronger than that of TiO_2_/SiNPs, which could be attributed to the C‐doping in C‐TiO_2_/SiNPs. Additionally, C‐TiO_2_/SiNPs (2.92 eV) possessed a narrowed E_g_ than that of TiO_2_/SiNPs (3.02 eV) as determined by the Tauc plot method (Figure 6D).

The semiconductor type and the corresponding flat‐band potential (E_fb_) were determined based on the Mott–Schottky measurement. As depicted in Figure 6E, the positive slopes were obtained both for C‐TiO_2_/SiNPs and TiO_2_/SiNPs, indicating that these materials possessed n‐type semiconductor traits.^[^ ^36^ ^]^ Meanwhile, the E_fb_ values (E_fb(Ag/AgCl)_) were measured as −1.39 and −1.66 eV (vs Ag/AgCl, pH = 7), respectively. Then, the E_fb_ values versus the normal hydrogen electrode (NHE) (E_fb(NHE)_) were calculated to be −1.193 and −1.463 eV, respectively, based on E_fb(NHE)_ = E_fb(Ag/AgCl)_ + 0.197 eV.^[^ ^36^ ^]^ In general, for n‐type semiconductor, its E_fb(NHE)_ value is approximately equivalent to the conduction band (CB) potential (E_CB_). Thus, according to E_g_ = E_VB_ − E_CB_,^[^ ^36^ ^]^ the valence band (VB) potential (E_VB_) values were separately calculated as 1.727 and 1.557 eV for C‐TiO_2_/SiNPs and TiO_2_/SiNPs. Based on the above results, the energy‐band diagrams of these two materials were depicted in Figure 6F. C‐TiO_2_/SiNPs possessed narrower E_g_, which was propitious to the visible light absorption and separation of holes (h^+^) and electrons (e^−^), and thus produced excellent photocatalytic activity. All the above results illustrated the superior photocatalytic activity of C‐TiO_2_/SiNPs to TiO_2_/SiNPs.

As known, the reactive oxidative species (ROS) may be produced in the presence of a photocatalyst under light irradiation to promote the catalytic process. Thus, herein, the ROS production ability of C‐TiO_2_/SiNPs was firstly evaluated by methyl orange assay.^[^ ^37^ ^]^ As shown in Figure S4 (Supporting Information), after C‐TiO_2_/SiNPs were subjected to the visible light irradiation for 30 min, the absorbance decreased, indicating the ROS production of C‐TiO_2_/SiNPs. To explain the contribution of reactive species generated by C‐TiO_2_/SiNPs in the photocatalytic process, radicals trapping experiments were further performed, using IPA (100 mM), 1,4‐benzoquinone (p‐BQ,1 mM) and ammonium oxalate (AO, 1 mM) as the scavengers for ⋅OH, O_2_^•−^ and h^+^, respectively. Compared to the degradation efficiency of OTC in the absence of any of these three scavengers (93.3%) (Figure 6G), the removal rates were reduced to 83.2%, 75.6% and 51.4% in the presence of IPA, AO and p‐BQ, respectively, revealing that all of them contributed to the photocatalytic degradation. Moreover, the generation of O_2_^•−^ and ⋅OH on C‐TiO_2_/SiNPs was verified using 5,5‐dimethyl‐1‐pyrroline *N*‐oxide (DMPO) spin‐trapping electron spin resonance (ESR) technique. Intuitively, the clear signal peaks corresponding to the photo‐generated O_2_^•−^ and ⋅OH were observed after light irradiation (Figure 6H,I),^[^ ^38^ ^]^ yet no signal in the dark condition, suggesting that C‐TiO_2_/SiNPs could effectively generate O_2_^•−^ and ⋅OH under visible light irradiation, which was consistent with the results from the radicals trapping experiment (Figure 6G).

#### 2.3.3 Transformation Pathway and Toxicity Assessment of Transformation Products of OTC

**Figure 7:**
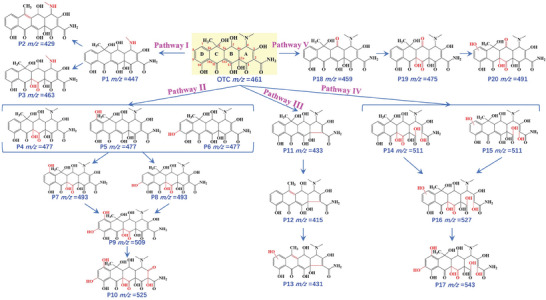
The proposed photodegradation pathways of OTC.

The photodegradation products of OTC were identified by an ultra‐performance liquid chromatography‐quadrupole time‐of‐flight mass spectrometry (UPLC‐Q‐TOF MS) to explore its transformation pathways. The MS spectra and detailed information about the transformation products are listed in Figure S5 (Supporting Information) and Table S2 (Supporting Information). All detected transformation products were deduced based on the corresponding mass spectra, fragment ions, and relevant published literatures.^[^ ^39^, ^40^, ^41^, ^42^, ^43^, ^44^, ^45^, ^46^, ^47^ ^]^ Five plausible transformation pathways of OTC were proposed (**Figure** **7**).

Pathway I: As reported,^[^ ^39^ ^]^ the methyl carbon in ‐N(CH_3_)_2_ group attached to C4 was the susceptible site attacked by electrophilic radical (h^+^). Consequently, P1 with the most intense signal at *m/z* 447 was formed by demethylation with the removal of one ‐CH_3_.^[^ ^40^, ^41^ ^]^ Then, P1 was transformed to P2 (*m/z* 429) by dehydration.^[^ ^42^ ^]^ Besides, similar to TC,^[^ ^43^ ^]^ the double bond and phenolic groups were easily attacked by ⋅OH, as the double bond was more reactive with radicals. Meanwhile, C11a‐C12 double bond with relatively higher electron density was more reactive than the C2═C3 double bond, due to the electron withdrawing effect of the amide group.^[^ ^43^, ^44^ ^]^ Thus, P3 (*m/z* 463) was generated as a result of hydroxylation of P2 at the C11a═C12 double bond.^[^ ^44^ ^]^

Pathway II: As mentioned in Pathway I, P4 (*m/z* 477) was obtained by hydroxylation of OTC at the C11a═C12 double bond. Moreover, ·OH could attack the *para* (C7) or *ortho* (C9) sites of the aromatic ring D for the higher electron density, generating P5 and P6 (*m/z* 477), respectively, in light of the localization effect of the phenolic hydroxyl group.^[^ ^43^, ^45^ ^]^ With further attack by ·OH at the same site, P7 and P8 (*m/z* 493) were generated.^[^ ^46^ ^]^ Meanwhile, multiple hydroxylated products, P9 (*m/z* 509) and P10 (*m/z* 525), were formed in a similar way.^[^ ^44^ ^]^

Pathway III: Initially, OTC experienced decarbonylation (−28 Da) to produce P11 (*m/z* 433).^[^ ^47^ ^]^ Then, P11 was transformed to P12 (*m/z* 415) via dehydration (−18 Da).^[^ ^40^, ^46^ ^]^ Further hydroxylation on the aromatic ring D resulted in the formation of P13 (*m/z* 431).^[^ ^40^ ^]^

Pathway IV: Ring‐opening reactions could also occur at the C1─C2 bond (sp^2^ hybridization) of OTC accompanying with the addition of two hydroxyl groups. With further attack of ⋅OH, hydroxyl addition to double bond at C11a═C12 and the aromatic ring D was supported by the detection of *m/z* 511, 527 and 543.^[^ ^44^, ^46^ ^]^

Pathway V: In this pathway, the secondary alcohol group at C5 in the B‐ring was oxidized to a ketone by the removal of 2H (−2 Da) under the attack of ⋅OH, i.e., quinonization, forming P18 (*m/z* 459). Afterward, P18 underwent hydroxylation at C11a‐C12 and the aromatic ring D was further attacked by ⋅OH, giving birth to P19 (*m/z* 475) and P20 (*m/z* 491), respectively.^[^ ^44^, ^46^ ^]^

To distinguish the isomers, the calculated log*P* (Clog*P*) values obtained from ChemDraw 12.0 were used. Generally, one compound with smaller Clog*P* value displayed shorter retention time and could be eluted earlier in a reversed‐phase chromatographic system.^[^ ^48^ ^]^ For example, a pair of isomers, P4 with Clog*P* of −3.003 and P5 with Clog*P* of −1.771, had retention time of 3.122 and 7.092 min, respectively.

Altogether, the transformation pathways of OTC typically included demethylation, dehydration, hydroxylation, decarbonylation, ring opening and secondary alcohol oxidation, based on the detected 20 transformation products (Table S2, Supporting Information) which have been previously reported during the photodegradation of OTC.^[^ ^39^, ^40^, ^41^, ^42^, ^43^, ^44^, ^45^, ^46^, ^47^ ^]^

The total toxicities of the transformation products were estimated by a luminous bacteria assay. I% represented the toxicity of the products to luminous bacteria, i.e., the greater of I%, the more toxic of the products. As shown in Figure S6 (Supporting Information), the I% values of the photocatalytic products of OTC were lower than that of OTC itself, demonstrating the lessened toxicity of the transformation products. In order to quantify the change scope in I%, (ΔI%)% was calculated (green column in Figure S6, Supporting Information). After treated with the C‐TiO_2_/SiNPs/LED system, (ΔI%)% of OTC was 73.7%, revealing that the photocatalytic degradation could significantly reduce the toxicity of OTC to luminous bacteria.

### 2.4 Combination of Fluorescence Detection and Photocatalytic Degradation

**Figure 8:**
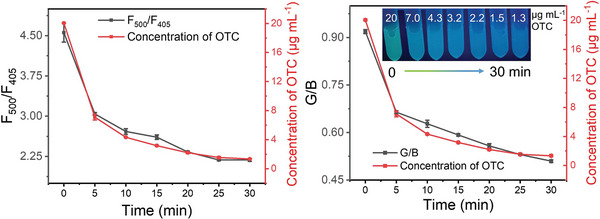
A) The dynamic change curves of signals of F_500_/F_405_ with degradation time (left), and the concentration of OTC determined by HPLC with degradation time (right) during OTC degradation process. B) The dynamic change curves of G/B value of photos with degradation time (left), and the concentration of OTC determined by HPLC with degradation time (right) during OTC degradation process. Inset: photos of the mixture solution under UV light irradiation (λ = 365 nm) at different time points.

As discussed in Section 2.2 and 2.3, C‐TiO_2_/SiNPs exhibited adorable performance for the detection and degradation of OTC. Expectedly, catalyzing the degradation of OTC and monitoring the degradation in real time could simultaneously be achieved using C‐TiO_2_/SiNPs as both the photocatalyst and fluorescence probe. The real‐time concentration of OTC in the degradation system was monitored by both the fluorescence signal (F_500_/F_405_) and HPLC at scheduled time points. As depicted in **Figure** **8**A, obviously, the concentration of OTC obtained by HPLC and the F_500_/F_405_ value in a relation to OTC concentration with the degradation time matched well to each other, implying the accuracy of fluorescence method.

Additionally, the fluorescence color of the degradation system changed from green to blue, as photographed in Figure 8B (Inset). Thus, to quickly evaluate the OTC levels in the degradation system, the fluorescence color could be adopted as a criterion. Furthermore, a mobile APP for treatment of pictures (Se Cai) was applied to read the red, green and blue (RGB) values of the fluorescence from the mixed solution at predetermined time intervals. As shown in Figure 8B, the concentration of OTC obtained by HPLC and the G/B value in a relation to OTC concentration with the degradation time also matched well to each other. The phenomenon implied that it was feasible to quickly determine the degree of degradation by taking photos using mobile phones, which would assist the assessment of degradation status quickly. Overall, both precise quantification by fluorescence and semi‐quantification by visual observation were faster and more convenient than traditional methods, i.e., HPLC. Based on the above results, C‐TiO_2_/SiNPs achieved dual goals, simultaneously monitoring by fluorescence/naked eye and photocatalysis by visible light.

## 3 Conclusion

In this study, a novel nanocomposite, C‐TiO_2_/SiNPs, consisting of SiNPs and C‐doped TiO_2_, were introduced and successfully used for simultaneous monitoring and degradation of OTC as a fluorescence probe and visible‐light catalyst. The detection mechanism involved IFE and AIE, i.e., the blue fluorescence of C‐TiO_2_/SiNPs was quenched by IFE and green fluorescence was turned on by AIE upon the addition of OTC. Hence, C‐TiO_2_/SiNPs was applied as a ratiometric fluorescence probe for OTC detection, achieving an LOD of 0.01 µg mL^−1^. Driven by superoxide radical, holes and hydroxyl radicals produced by C‐TiO_2_/SiNPs under visible light irradiation, up to 93.3% of OTC was removed only in 30 min. What's more, the total toxicities of the transformation products of OTC were attenuated, which was beneficial for environmental remediation. At last, the capability of C‐TiO_2_/SiNPs to simultaneously monitor and remove OTC was confirmed. The concentration changes of OTC could be observed by the naked eye through altered fluorescence color, as well as accurately quantified by fluorescence spectrometry. Overall, this work presents an innovative “two birds, one stone” strategy for designing a bifunctional nanocomposite that can be used simultaneously for the detection and removal of environmental pollutants.

## 4 Experimental Section

### Materials

Titanium tetraisopropanolate (TPT), APTES, nitric acid (HNO_3_), EtOH, NaCl, IPA, MeOH, ACN, DMSO, p‐BQ, AO, sodium sulfate (Na_2_SO_4_), barium sulfate (BaSO_4_), hydrochloric acid (HCl), humic acid, sodium bicarbonate, trisodium phosphate, potassium nitrate and sodium hydroxide were all bought from Sinopharm Chemical Reagent Co., Ltd. (Shanghai, China). Ascorbic acid, DOC, TC, OTC, chloramphenicol, roxithromycin, lincomycin, dopamine, glucose, L‐tryptophan, D‐tryptophan, glutathione, ibuprofen, DMPO, potassium ferricyanide and potassium ferrocyanide were supplied from Aladdin Chemical Reagent Co., Ltd. (Shanghai, China). Nafion117 was purchased from Sigma‐Aldrich (USA). The lyophilized bright luminescent bacillus powder was obtained from Shang Hai Bao Cang Center (Shanghai, China). Ultrapure water was prepared through a Heal Fore NW system (Shanghai, China).

### Instruments

SEM image was captured by a TESCAN MIRA 3 scanning electron microscope (TESCAN Brno, s.r.o., Czech Republic). The XRD patterns were obtained on an XRD‐6100 diffractometer (Shimadzu, Japan) from 10° to 80° at 5°/min. XPS measurements were carried out on a Thermo Escalab 250Xi spectrometer (Thermo, USA). EA was performed by an Elementar UNICUBE elemental analyzer (Elementar, Germany). A fluorescence lifetime spectrometer with Felix 32 system (Photon Technology International, Canada) was applied to record the fluorescence lifetime decay curves. An A200 Bruker electron spin (paramagnetic) resonance spectrometer (Bruker, Germany) was used to study the O_2_^•−^ and ·OH generated by C‐TiO_2_/SiNPs. The fluorescence measurement was obtained using an F‐4600 fluorescence spectrometer (Hitachi, Japan). UV‐vis DRS were obtained on an UV‐3600 UV‐Vis‐NIR spectrophotometer (Shimadzu, Japan), using BaSO_4_ as the reflectance standard. A CHI660D electrochemical workstation equipped with a representative three‐electrode system (ChenHua Instruments, China) was applied to measure EIS and transient photocurrent response with a conventional three‐electrode system.

### Synthesis of C‐TiO_2_/SiNPs

First, the C‐TiO_2_ precursor was prepared by heating 30 mL of the mixture of water and IPA (1:1, v: v) containing 30 mg of ascorbic acid, 1.5 mL of HNO_3_ and 5 mL of TPT at 80°C with stirring for 1 h. In this process, ascorbic acid served as the C‐doping reagent, TPT was the TiO_2_ source, the mixture of water and IPA provided a suitable condition for TPT hydrolysis, and HNO_3_ could stabilize the C‐TiO_2_ precursor.^[^ ^49^ ^]^ Additionally, for comparison, the TiO_2_ precursor was prepared without the addition of ascorbic acid through the same process.

Then, 1.5 mL APTES and 1.5 mL H_2_O were mixed with 3 mL of the C‐TiO_2_ precursor, and transferred to a Teflon lined stainless steel autoclave, subjecting to heating at 180°C for 6 h. The obtained solid, named as C‐TiO_2_/SiNPs, was washed by copious deionized water for three times for further use. In a similar way, several control materials, TiO_2_, C‐TiO_2_, SiNPs and TiO_2_/SiNPs, were prepared as listed in Table S3 (Supporting Information) under the same hydrothermal condition.

### Fluorescence Detection of OTC Using C‐TiO_2_/SiNPs as the Probe

To detect OTC, the C‐TiO_2_/SiNPs dispersion solution was added to the tested OTC aqueous solutions with incubation for the prescribed time. The fluorescence intensities, i.e., F_405_ and F_500_, were measured at emission wavelengths of 405 nm and 500 nm, respectively, both excited at 365 nm, and utilized to quantify the concentration of OTC. The detection conditions, including the concentration of C‐TiO_2_/SiNPs, incubation time, NaCl concentration and pHs, were optimized using F_500_/F_405_ as the criterion.

Under the optimal detection conditions, the linearity between the value of F_500_/F_405_ and the concentration of OTC was plotted. LOD was calculated based on the IUPAC method (3σ/k, σ was the standard deviation of F_500_/F_405_ without OTC, and k was the slope of the calibration curve).^[^ ^50^ ^]^

To test the detection selectivity of C‐TiO_2_/SiNPs against OTC, a series of possible interference substances were investigated, including DOC, TC, OTC, chloramphenicol, roxithromycin, lincomycin, dopamine, glucose, L‐tryptophan, D‐tryptophan, glutathione, and ibuprofen. The fluorescence intensities of the mixtures of C‐TiO_2_/SiNPs (400 µg mL^−1^) and individual substance (50 µg mL^−1^) were measured under the optimal detection conditions. In the coexisting effect evaluation tests, the concentration of OTC was kept at 5 µg mL^−1^.

For the visual detection, the incubated solution with C‐TiO_2_/SiNPs and OTC was subjected to a camera obscura with UV light (λ = 365 nm), and the fluorescence color was observed and photographed.

### Photoelectrochemical Measurements

In 0.1 M Na_2_SO_4_ electrolyte (pH 7.0) or 5 mM of potassium ferricyanide/potassium ferrocyanide (EIS measurement), an indium tin oxide (ITO) electrode deposited with the tested materials was used as the working electrode, while an Ag/AgCl electrode and a platinum foil electrode were employed as reference and counter electrode, respectively. To prepare the working electrode, 10 mg mL^−1^ of the tested materials (e.g., C‐TiO_2_/SiNPs and TiO_2_/SiNPs) were dispersed in 1 mL of 50% EtOH, followed by the addition of 20 µL of Nafion117 with sonication for 30 min to form a uniform slurry. A volume of 100 µL of this slurry was dropped onto the surface of the ITO electrode and dried in an oven. An LED lamp (λ = 420 nm) was utilized as the light source for the transient photocurrent response test.

### Photocatalytic Degradation of OTC

In a typical experiment, 30 mL of OTC solution (20 µg mL^−1^) with 400 µg mL^−1^ of C‐TiO_2_/SiNPs was subjected to LED light equipped with a polarizing film for filtering out the UV light. Under continuous visible light irradiation and magnetic stirring, 1 mL of the suspension was sampled at an interval of 5 min and centrifuged at 12000 rpm for 5 min. The supernatant was then collected and injected to HPLC for determining the concentration of OTC, as described in Text S2 (Supporting Information). The identification of transformation products of OTC by UPLC‐Q‐TOF MS was described in Text S3 (Supporting Information).

The removal rate (%) and the catalytic rate (*k*) based on quasi‐first‐order kinetic model were calculated according to the Equation (1) and Equation (2), respectively:

(1) $$\mathrm{Removal}\;\mathrm{rate}\;(\%)=\frac{C_{0}-C_{t}}{C_{0}}\times 100\%$$

(2) $$\mathrm{Ln}\;(\frac{C_{t}}{C_{0}})=-\mathrm{kt}$$

where C_0_ was the initial concentration of OTC, C_t_ was the OTC concentration at the time t (min).

### Toxicity Assessment

To test the total toxicity of transformation products of OTC, the OTC solution (20 µg mL^−1^) photo‐catalyzed by C‐TiO_2_/SiNPs for 30 min, which was supposed to be mostly transformed according to the kinetics result of OTC, was diluted 10 times with 3% NaCl solution, i.e., 0.2 mL of degradation solution was mixed with 1.8 mL of 3% NaCl solution. Then, 20 µL of the resuscitated bacterial solution was added to this diluted solution with fully agitation for exact 15 min under room temperature. The luminous intensities of these solutions were determined (λ_ex_ = 280 nm, λ_em_ = 480 nm). The luminous intensity inhibition rate (I%) and percentage change of I% ((ΔI%)%) were calculated by Equation (3) and Equation (4), respectively.

(3) $$I\%=\frac{I_{0}-I_{X}}{I_{0}}\times 100\%$$

(4) $$\left(\Delta I\%\right)\%=\frac{I_{i}\%-I_{P}\%}{I_{i}\%}\times 100\%$$

I_0_ and I_X_ represent the luminous intensities of the pure 3% NaCl solution and the sample solution, respectively. I_i_% and I_P_% represent the luminous intensity inhibition rates of OTC degradation solution at the initial time and ending time, respectively.

### Statistics and Reproducibility

In this work, n ≥ 3 independent samples were prepared in parallel for each quantitative statistical analysis to ensure the reproducibility. The data were presented as the mean ± standard deviation (S.D.). The one‐way analysis of variance (ANOVA) was calculated by Origin 2024. * or ^#^ *p* < 0.05, ** or ^##^ *p* < 0.01, *** or ^###^ *p* < 0.001 indicated the differences between the measured data were significant.

## Conflict of Interest

The authors declare no conflict of interest.

## Authorship Contributions

L.M. and Z.L. contribute to this work equally. L.M. provided resources, data analysis and paper drafting; Z.L. performed data acquisition, analysis and organization, initial paper drafting; Y.S. performed data acquisition, analysis; Y.X. performed data acquisition; M.J. supervised the project and edited the paper; X.Y. supervised the project and edited the paper; L.X. supervised the project, provided resources, finalized data analysis and edited the paper.

## Supporting information



Supporting Information

## Data Availability

The data that support the findings of this study are available from the corresponding author upon reasonable request.
