# Supplementary material for: Two Birds With One Stone: A Novel Nanocomposite Combining Silicon Nanoparticles and Carbon‐Doped Titania as the Fluorescence Probe and Visible Light Photocatalyst for Simultaneous Detection and Removal of Oxytetracycline
Source: Adv Sci (Weinh). 2024 Dec 9;12(5):2410306. doi: 10.1002/advs.202410306 (PMC11791936; doi:10.1002/advs.202410306)
Supplement: Supplementary file 1 — Supporting Information [file ADVS-12-2410306-s001.docx]

**Supporting Information**

**Two Birds with One Stone: A Novel Nanocomposite Combining Silicon Nanoparticles and Carbon-Doped Titania as the Fluorescence Probe and Visible Light Photocatalyst for Simultaneous Detection and Removal of Oxytetracycline**

Liyun Ma^a, #^, Zhi Li^b, #^, Yiyi Shi^b^, Yuchen Xie^b^, Ming Jiang^b, *^, Xu Yu^b, *^, Li Xu^b, *^

^a^ Department of Pharmacy, Union Hospital, Tongji Medical College, Huazhong University of Science and Technology, Wuhan 430022, PR China

^b^ Tongji School of Pharmacy, Huazhong University of Science and Technology, Wuhan 430030, PR China

**Text S1 The Stern-Volmer equation**

The Stern-Volmer equation is as below:

$$\frac{\text{F}_{\text{0}}}{\text{F}}\text{=1+}\text{K}_{\text{SV}}\left[ \text{OTC} \right]\text{ }\text{ }\text{ }$$

where F_0_ and F represent the fluorescence intensity of one probe in the presence and absence of OTC, respectively; [OTC] represents the concentration of OTC; K_SV_ is the Stern-Volmer constant.

**Text S2 Determination of OTC by HPLC**

The concentration of OTC was determined on an LC-40AT high performance liquid chromatography (HPLC, Shimadzu Co., Japan). A DIONEX ODS column (2.1 mm×150 mm, 3 μm) was used for separation with the column temperature of 50°C. The mobile phase was the mixture of 10% organic phase (methanol: acetonitrile= 2:1, v: v) and 90% oxalic acid solution (0.01 M) at a flow rate of 0.35 mL/min. The injection volume was 10 μL. The detection wavelength was set as 350 nm.

**Text** **S3 Identification of** **transformation** **products** **of OTC** **by UPLC-Q-TOF MS**

The transformation products of OTC were identified by a UPLC-Q-TOF MS (ACQUITY UPLC/Xevo G2XS Q-TOF MS, Waters, USA). The separation was conducted on a DIONEX ODS column (2.1 mm×150 mm, 3 μm) with a column temperature of 40°C. The injection volume was 1 μL. The mobile phase was the same with that used in HPLC (Text S2) at a flow rate of 0.2 mL/min. The mass spectrum was obtained in positive and sensitivity modes with the mass range of 50-1200 (*m/z*). Other MS conditions, including the capillary voltage of 2.5 kV, sampling cone of 40 V, source temperature of 120°C, desolvation temperature of 400°C, cone gas flow of 50 L/h, desolvation gas flow of 800 L/h and trap gas flow of 2.0 mL/min, were used.

**Table S1** The outcome of elemental analysis

| Element  Materials | C (%) | N (%) | O (%) | H (%) |
| --- | --- | --- | --- | --- |
| TiO_2_ | 0.74 | 0.24 | 2.20 | 0.76 |
| C-TiO_2_ | 1.23 | 0.21 | 5.00 | 1.25 |
| TiO_2_/SiNPs | 8.01 | 2.63 | 2.53 | 7.54 |
| C-TiO_2_/SiNPs | 8.65 | 3.32 | 2.75 | 6.93 |

**Table S2** TOC and its transformation products detected by UPLC-Q-TOF MS

| **Compound** | **Retention time (min)** | **[M+H]^+^ Experimental mass** | **Chemical**  **formula** | **Exact mass** | **[M+H]^+^ Exact mass** | **Relative mass error (ppm)** | **Fragment ion** | **Probable structure** |
| --- | --- | --- | --- | --- | --- | --- | --- | --- |
| OTC | 4.804 | 461.1605 | C_22_H_24_N_2_O_9_ | 460.1482 | 461.1560 | 9.8 | 443.1449, 426.1175 |  |
| P1 | 1.921 | 447.1375 | C_21_H_22_N_2_O_9_ | 446.1325 | 447.1404 | -6.5 | 430.1140 |  |
| P2 | 4.041 | 429.1334 | C_21_H_20_N_2_O_8_ | 428.1220 | 429.1298 | 8.4 | 412.1021 |  |
| P3 | 2.649 | 463.1358 | C_21_H_22_N_2_O_10_ | 462.1274 | 463.1353 | 1.1 | 447.1375, 429.1252 |  |
| P4^#^ | 3.122 | 477.1492 | C_22_H_24_N_2_O_10_ | 476.1431 | 477.1509 | -3.6 | 463.1358, 447.1375 |  |
| P5^#^ | 7.092 | 477.1492 | C_22_H_24_N_2_O_10_ | 476.1431 | 477.1509 | -3.6 | 461.1605, 443.1449 |  |
| P6^#^ | 4.359 | 477.1492 | C_22_H_24_N_2_O_10_ | 476.1431 | 477.1509 | -3.6 | 461.1521, 447.1375, 429.1334 |  |
| P7^##^ | 2.787 | 493.1499 | C_22_H_24_N_2_O_11_ | 492.1380 | 493.1458 | 8.3 | 477.1492, 463.1358, 447.1375 |  |
| P8^##^ | 2.007 | 493.1412 | C_22_H_24_N_2_O_11_ | 492.1380 | 493.1458 | -9.3 | 447.1375, 430.1140 |  |
| P9 | 1.938 | 509.1409 | C_22_H_24_N_2_O_12_ | 508.1329 | 509.1408 | 0.2 | 493.1412, 463.1358 |  |
| P10 | 2.499 | 525.1362 | C_22_H_24_N_2_O_13_ | 524.1278 | 525.1357 | 1.0 | 509.1409, 493.1412, 463.1358, 447.1375 |  |
| P11 | 4.174 | 433.1602 | C_21_H_24_N_2_O_8_ | 432.1533 | 433.1610 | -1.8 | 416.1362 |  |
| P12 | 3.902 | 415.1477 | C_21_H_22_N_2_O_7_ | 414.1427 | 415.1505 | -6.7 | 398.1183 |  |
| P13 | 3.666 | 431.1444 | C_21_H_22_N_2_O_8_ | 430.1376 | 431.1454 | -2.3 | 429.1252 |  |
| P14^###^ | 2.123 | 511.1544 | C_22_H_26_N_2_O_12_ | 510.1486 | 511.1564 | -3.9 | 493.1412, 447.1375 |  |
| P15^###^ | 2.412 | 511.1544 | C_22_H_26_N_2_O_12_ | 510.1486 | 511.1564 | -3.9 | 493.1412, 447.1375 |  |
| P16 | 2.464 | 527.1541 | C_22_H_26_N_2_O_13_ | 526.1435 | 527.1513 | 5.3 | 493.1412, 447.1375 |  |
| P17 | 1.701 | 543.1451 | C_22_H_26_N_2_O_14_ | 542.1384 | 543.1462 | -2.0 | 525.1362 |  |
| P18 | 3.481 | 459.1392 | C_22_H_22_N_2_O_9_ | 458.1325 | 459.1404 | -2.6 | 431.1444, 402.1148 |  |
| P19 | 5.445 | 475.1357 | C_22_H_22_N_2_O_10_ | 474.1274 | 475.1353 | 0.8 | 461.1605 |  |
| P20 | 6.023 | 491.1289 | C_22_H_22_N_2_O_11_ | 490.1224 | 491.1302 | -2.6 | 475.1357, 461.1521 |  |

^#, ##, ###^ refers to three sets of isomers respectively.

**Table S3** The reactant, preparation condition and shorten name for the materials

| Reactant | Preparation condition | Shorten Name |
| --- | --- | --- |
| TPT, HNO_3_, IPA, H_2_O | Water bath, stirring, 80℃, 1 h | TiO_2_ precursor |
| TPT, HNO_3_, IPA, H_2_O, ascorbic acid | Water bath, stirring, 80℃, 1 h | C-TiO_2_ precursor |
| TiO_2_ precursor, H_2_O | Hydrothermal, 180℃, 6 h | TiO_2_ |
| C-TiO_2_ precursor, H_2_O | Hydrothermal, 180℃, 6 h | C-TiO_2_ |
| APTES, H_2_O | Hydrothermal, 180℃, 6 h | SiNPs |
| TiO_2_ precursor, APTES, H_2_O | Hydrothermal, 180℃, 6 h | TiO_2_/SiNPs |
| C-TiO_2_ precursor, APTES, H_2_O | Hydrothermal, 180℃, 6 h | C-TiO_2_/SiNPs |


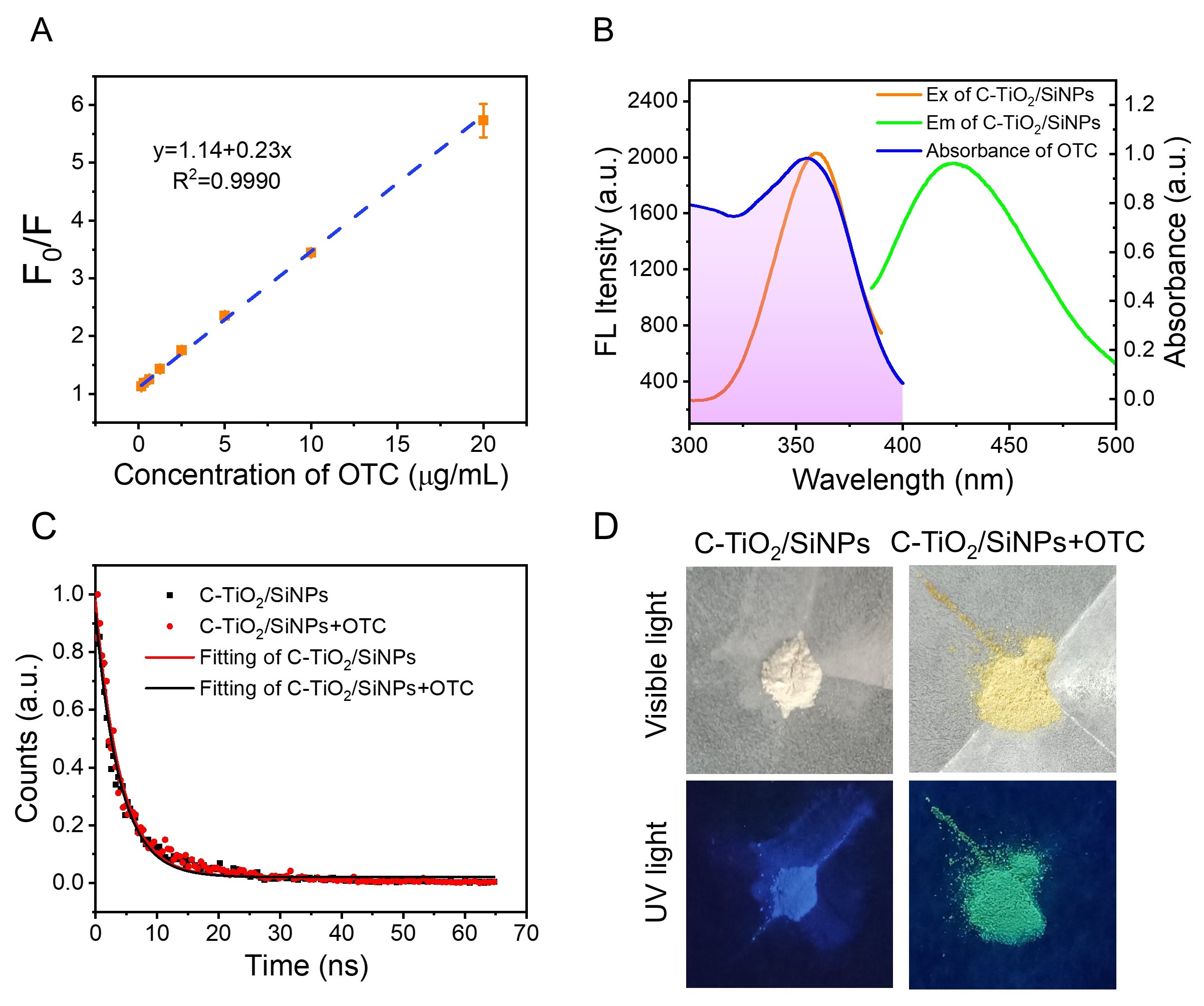


**Figure S1** (A) The Stern-Volmer fitting plot at OTC concentrations of 0.078-20 μg/mL. (B) Overlay of the fluorescence spectra of C-TiO_2_/SiNPs and UV-vis spectrum of OTC. (C) The fluorescence lifetime decay curves of C-TiO_2_/SiNPs (400 μg/mL) in the absence and presence of OTC (5 μg/mL) (λ_ex_=365 nm, λ_em_ = 430 nm). (D) Photos of C-TiO_2_/SiNPs and C-TiO_2_/SiNPs + OTC under visible and UV light (λ = 365 nm).


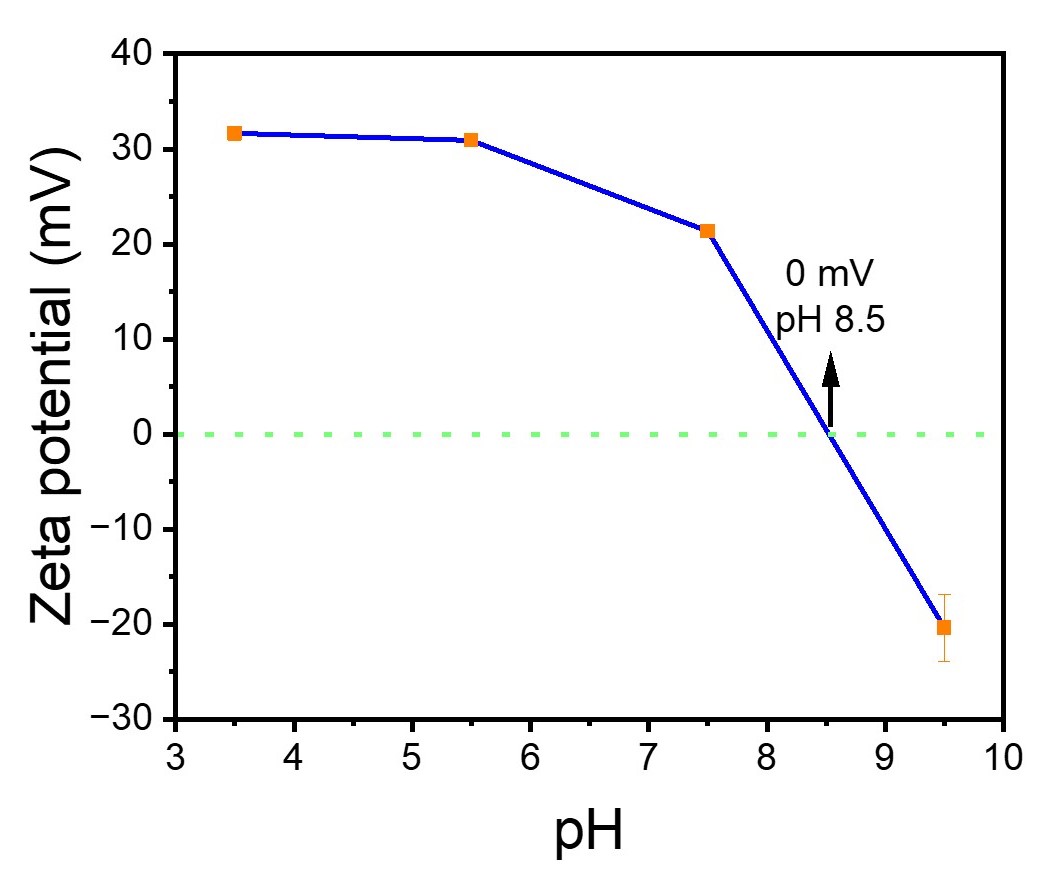


**Figure S2** The Zeta potential of C-TiO_2_/SiNPs in different pHs.


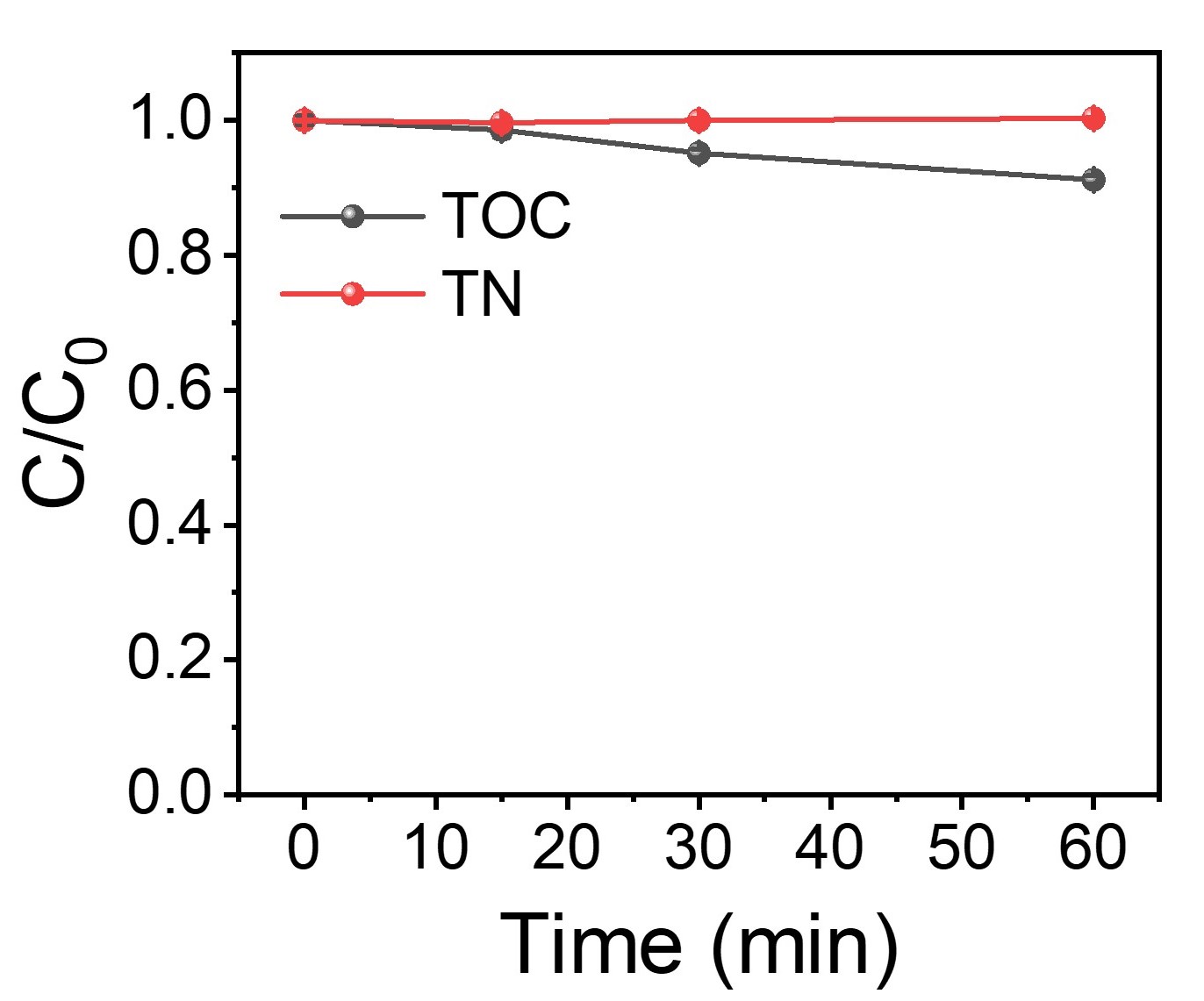


**Figure S3** The TOC and TN contents during the visible light photodegradation of OTC by C-TiO_2_/SiNPs.


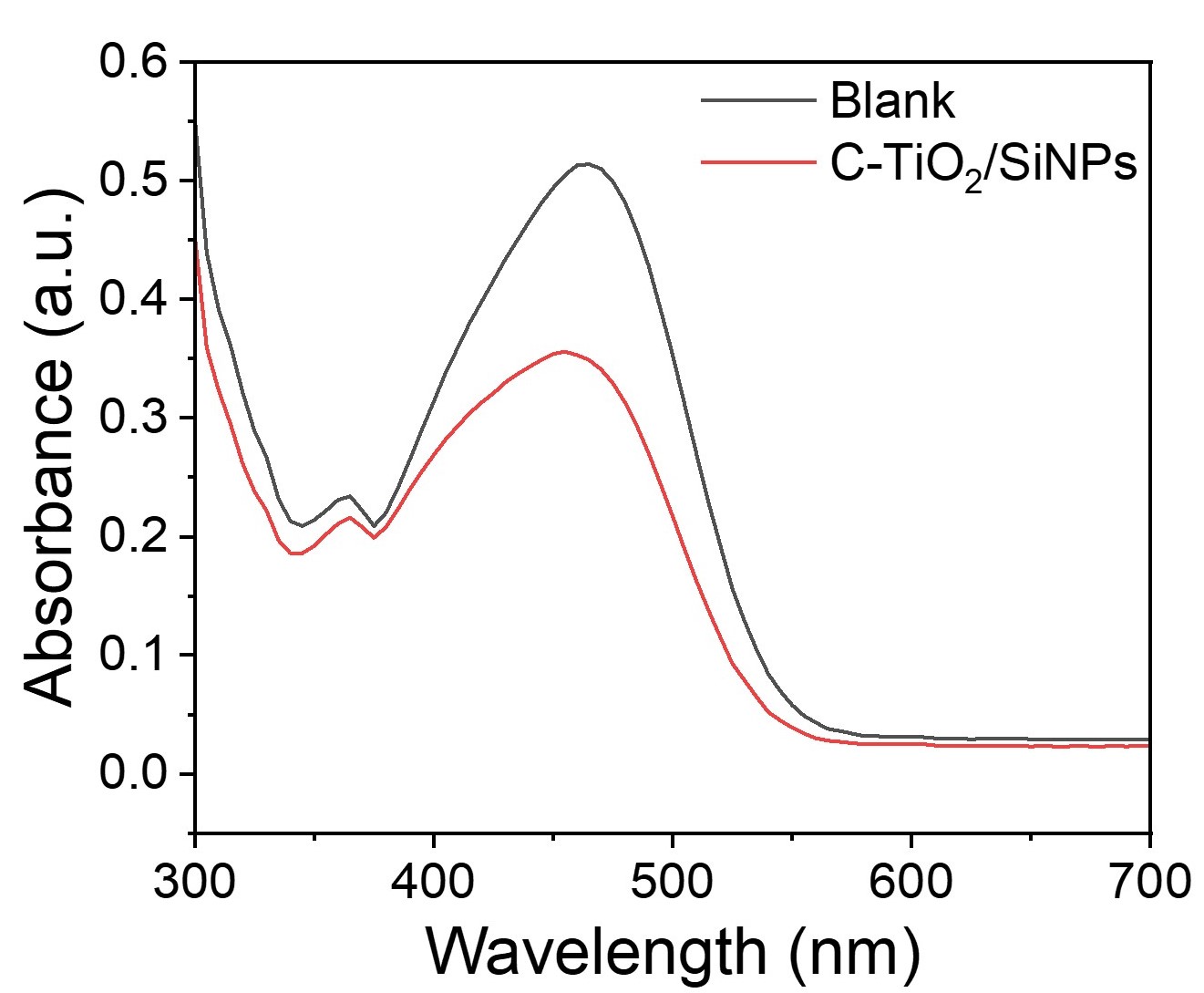


**Figure S4** The UV absorption spectra of methyl orange with and without C-TiO_2_/SiNPs after visible light irradiation of 30 min (C-TiO_2_/SiNPs: 1.0 mg/mL, methyl orange: 0.05 mM).


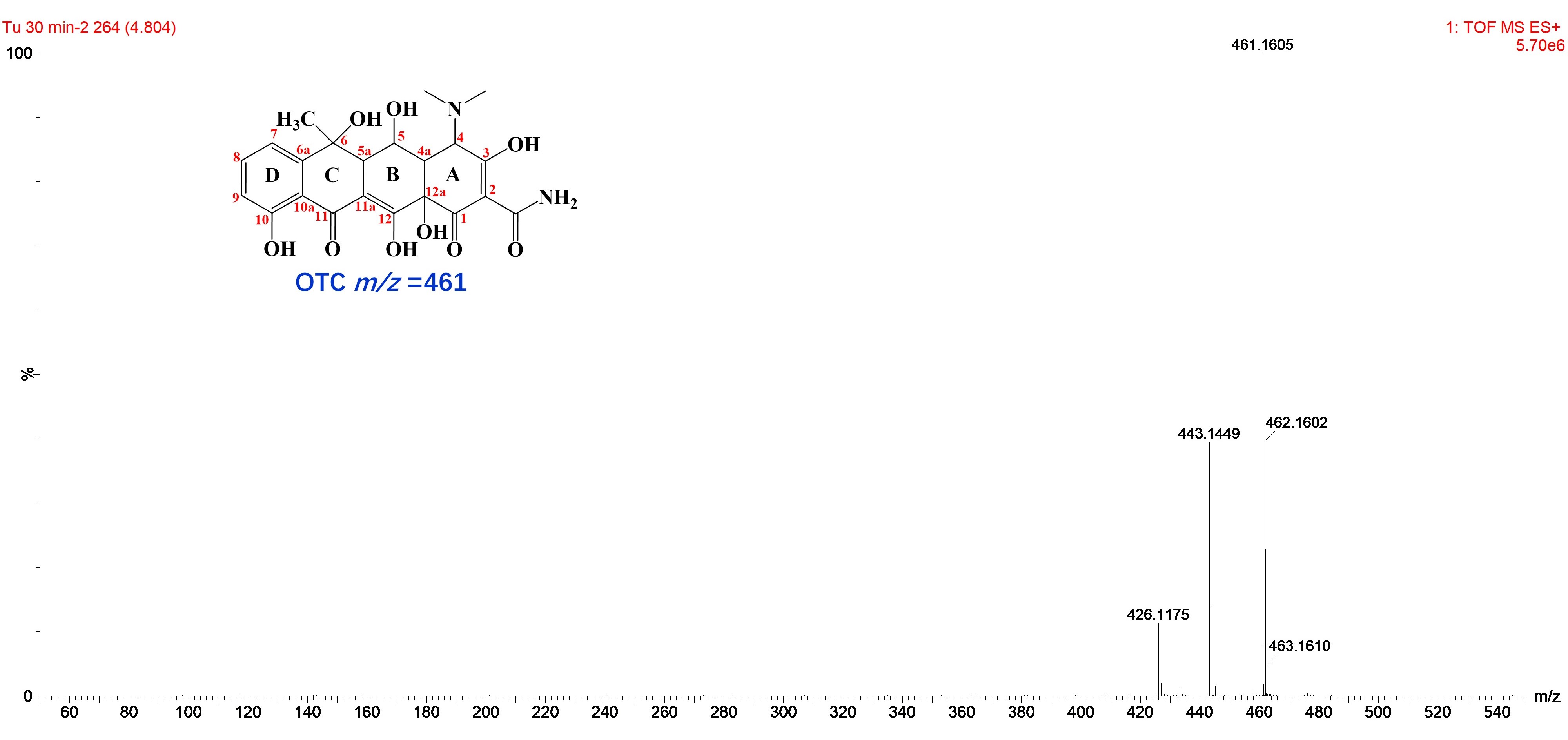

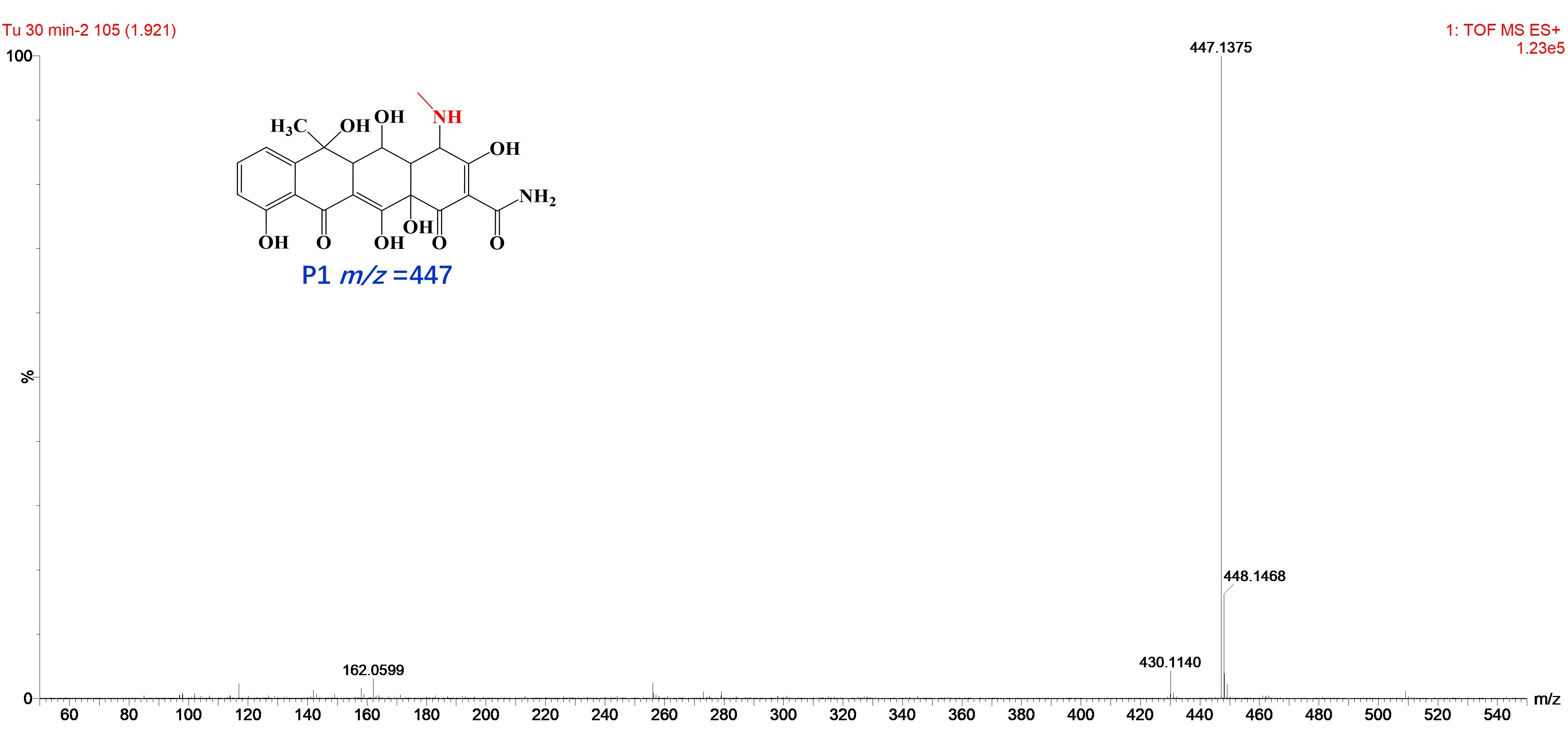

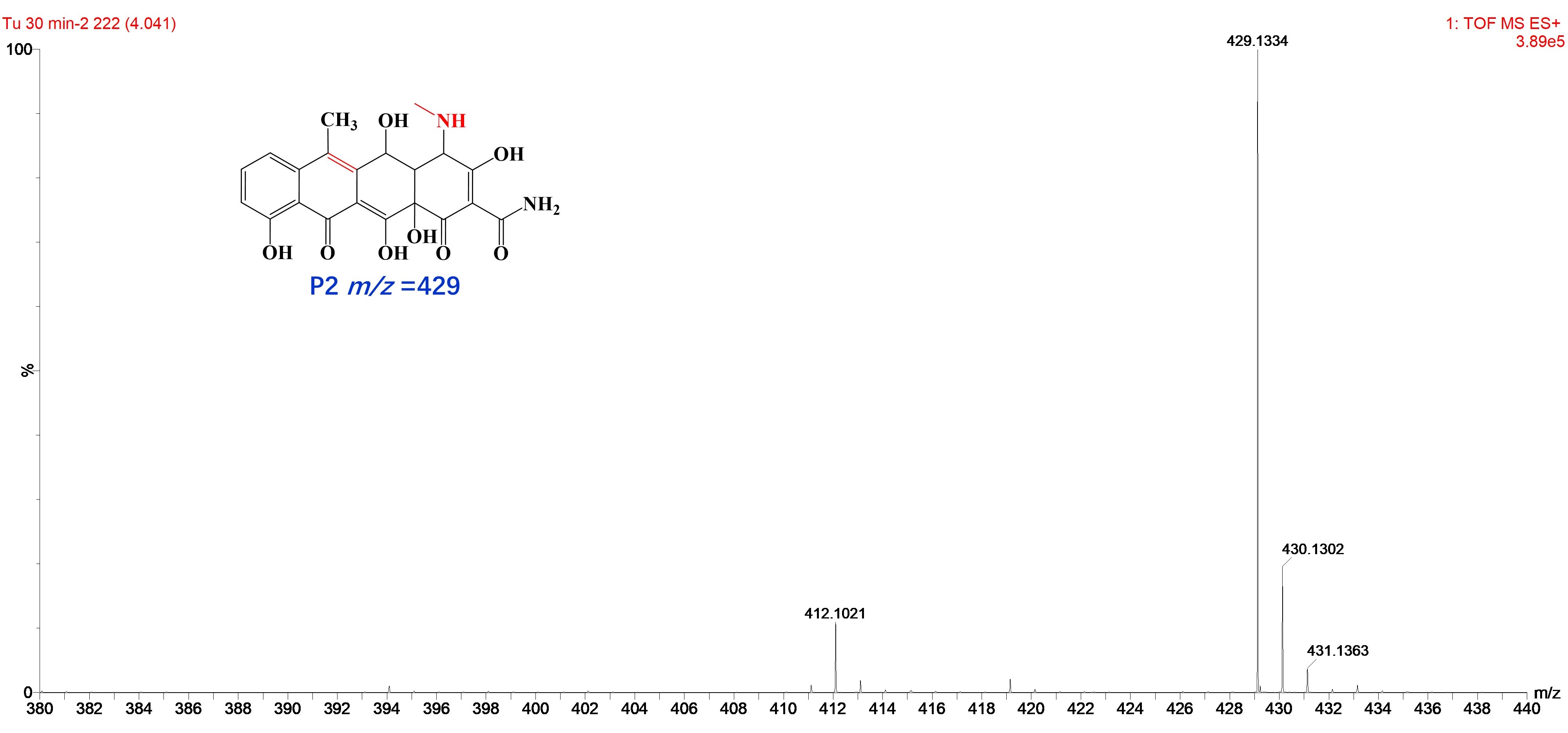

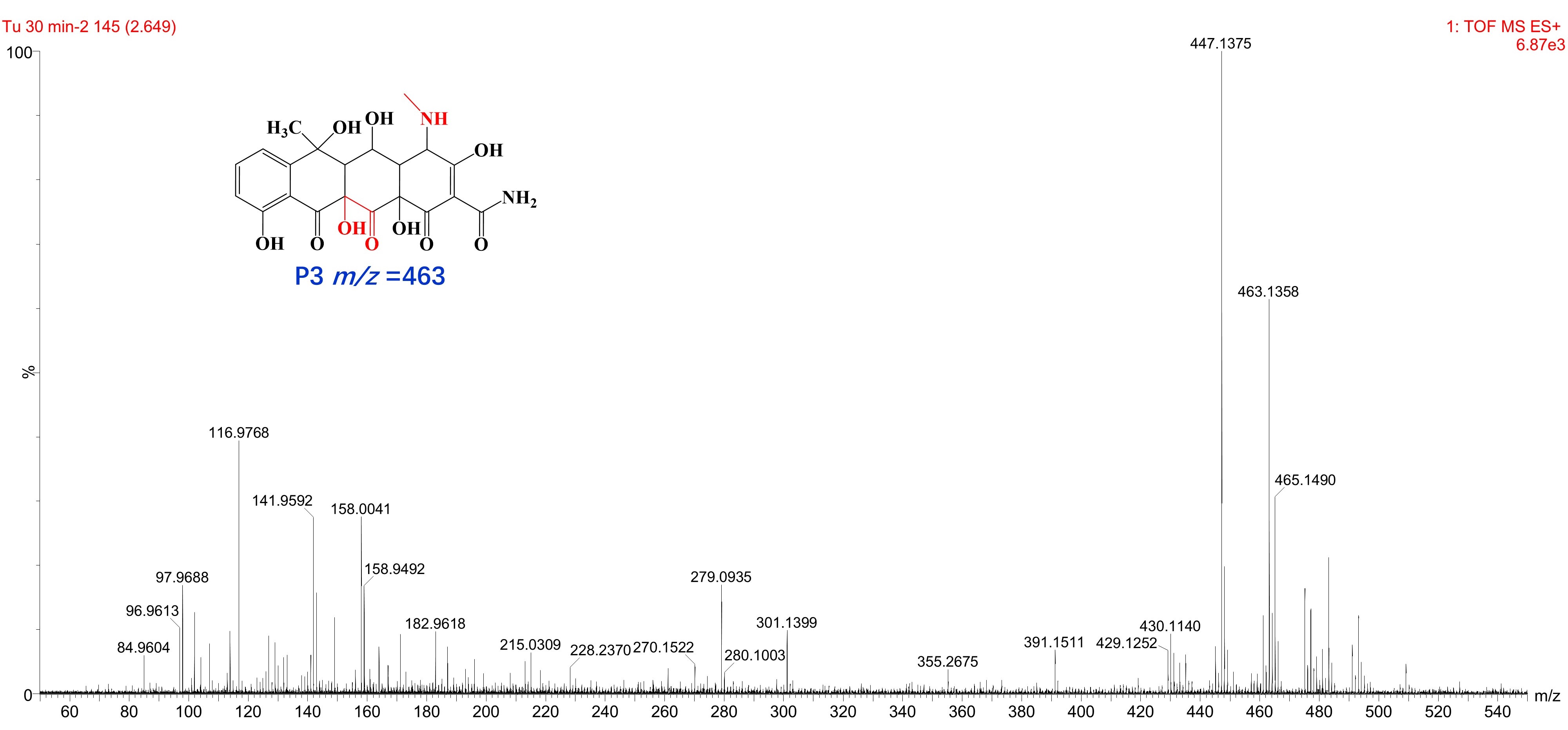

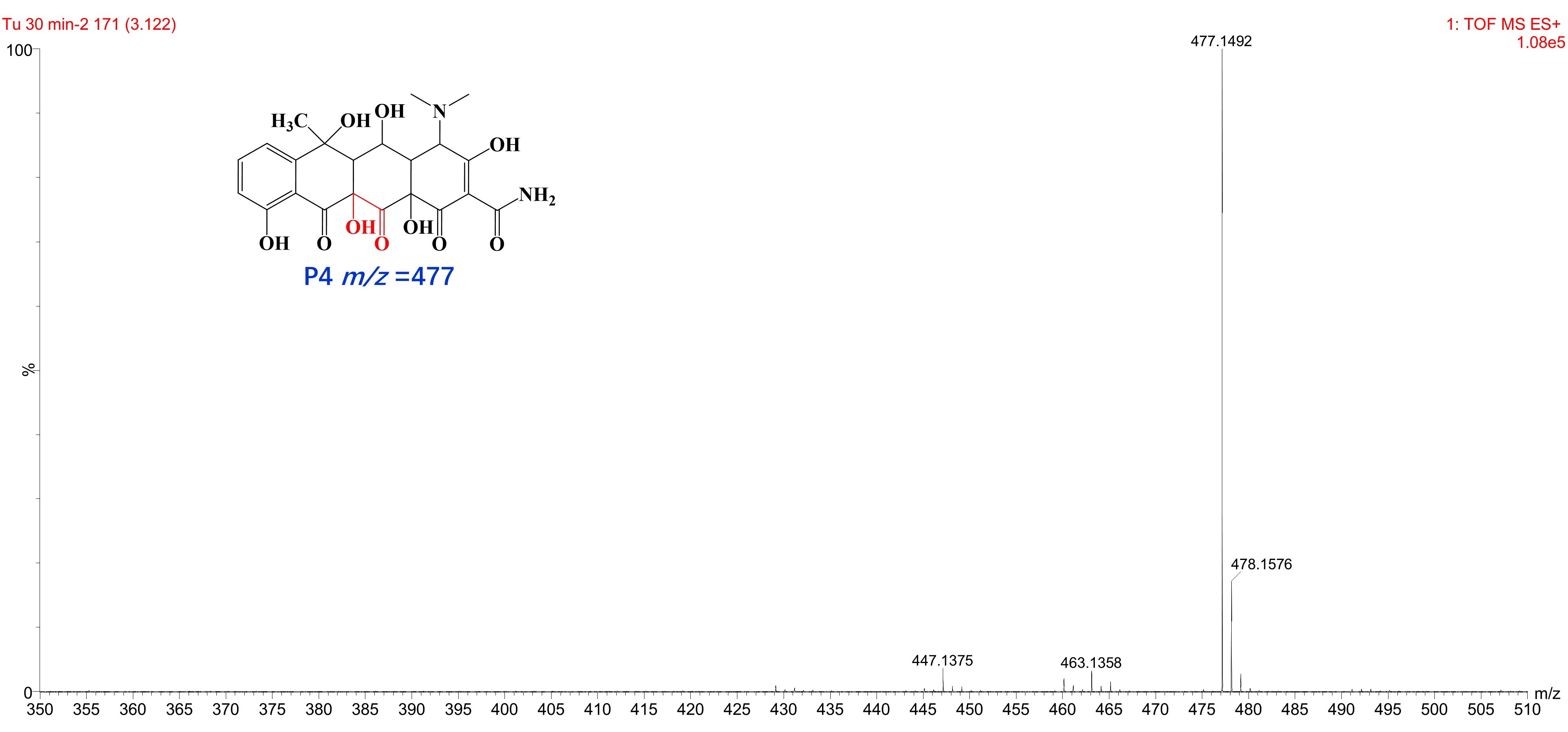

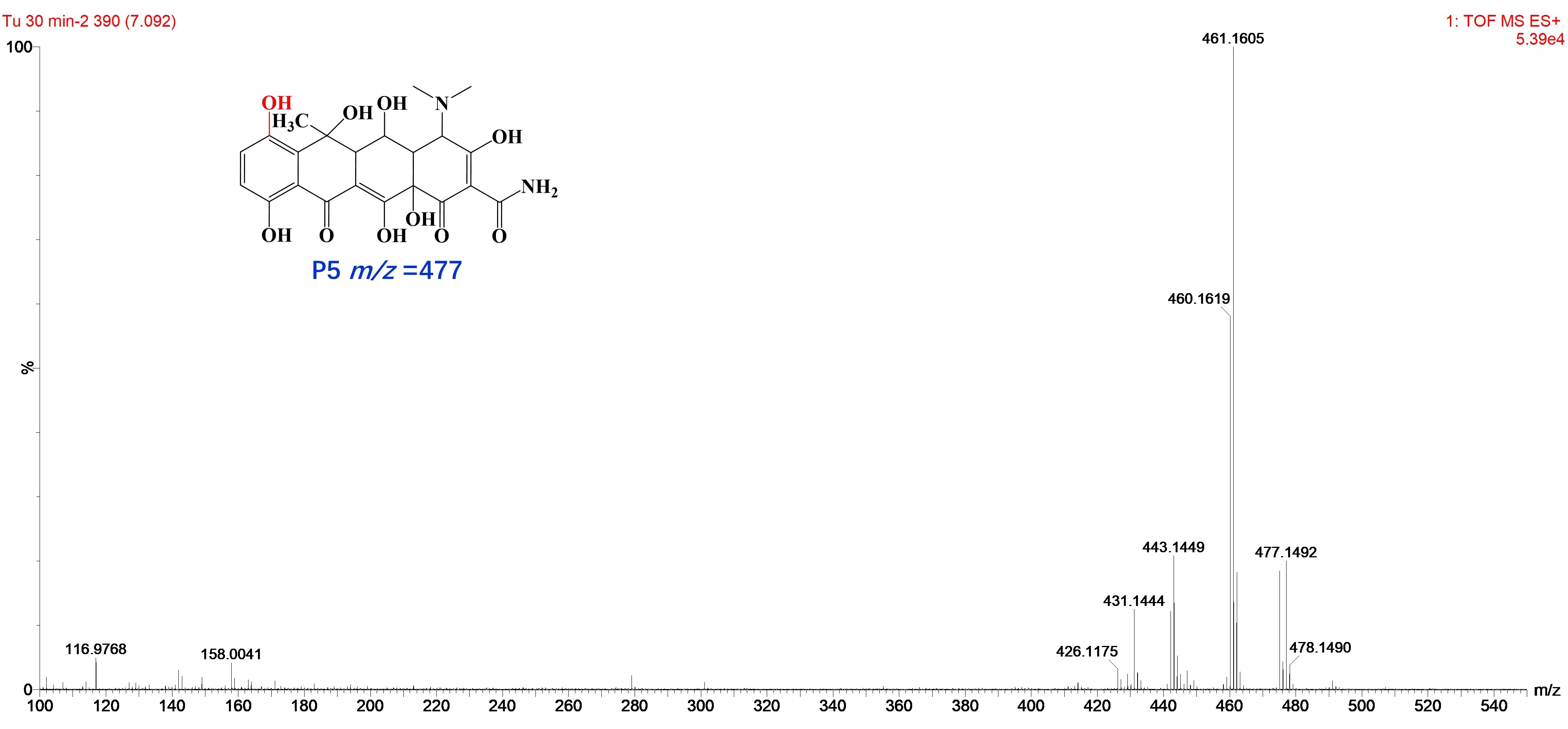

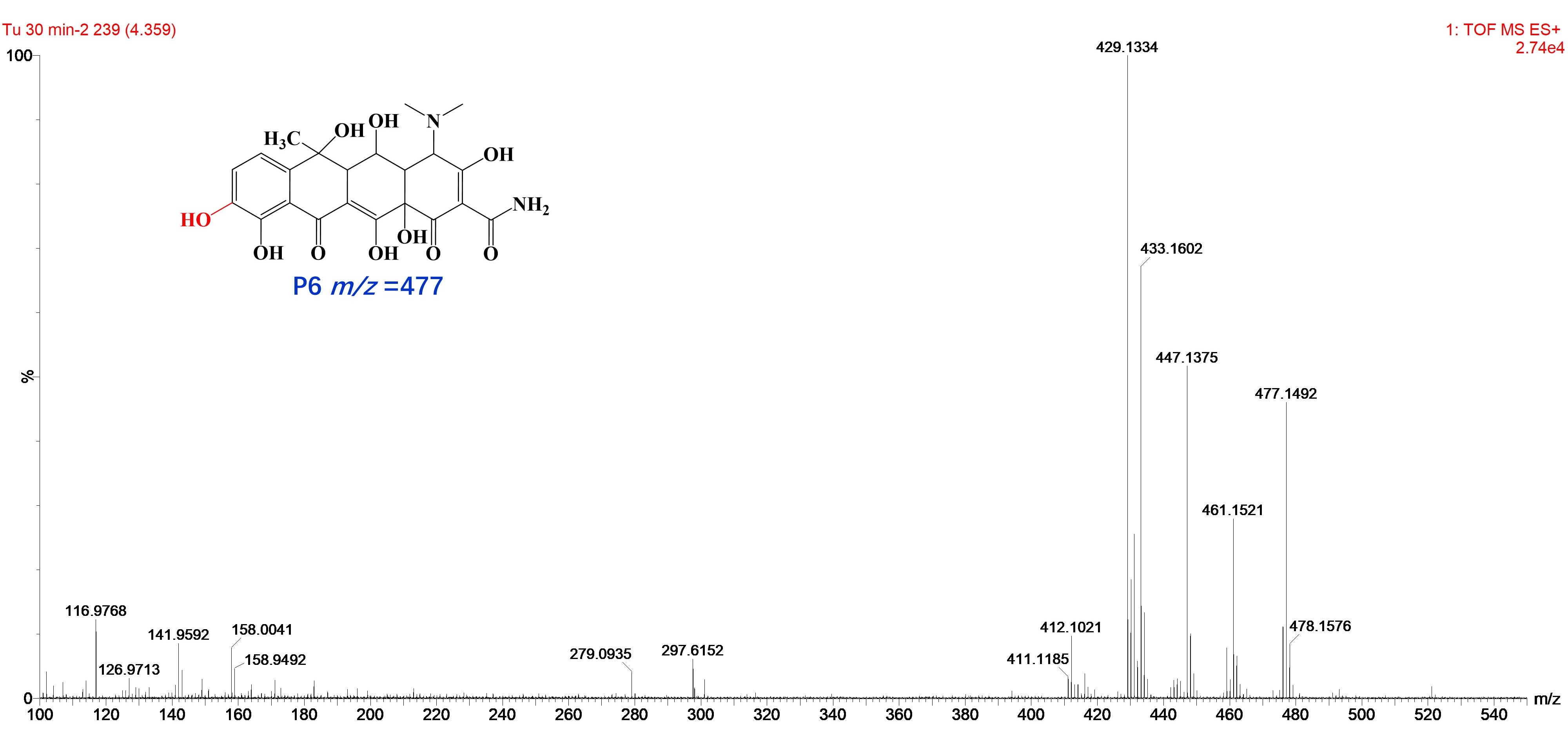

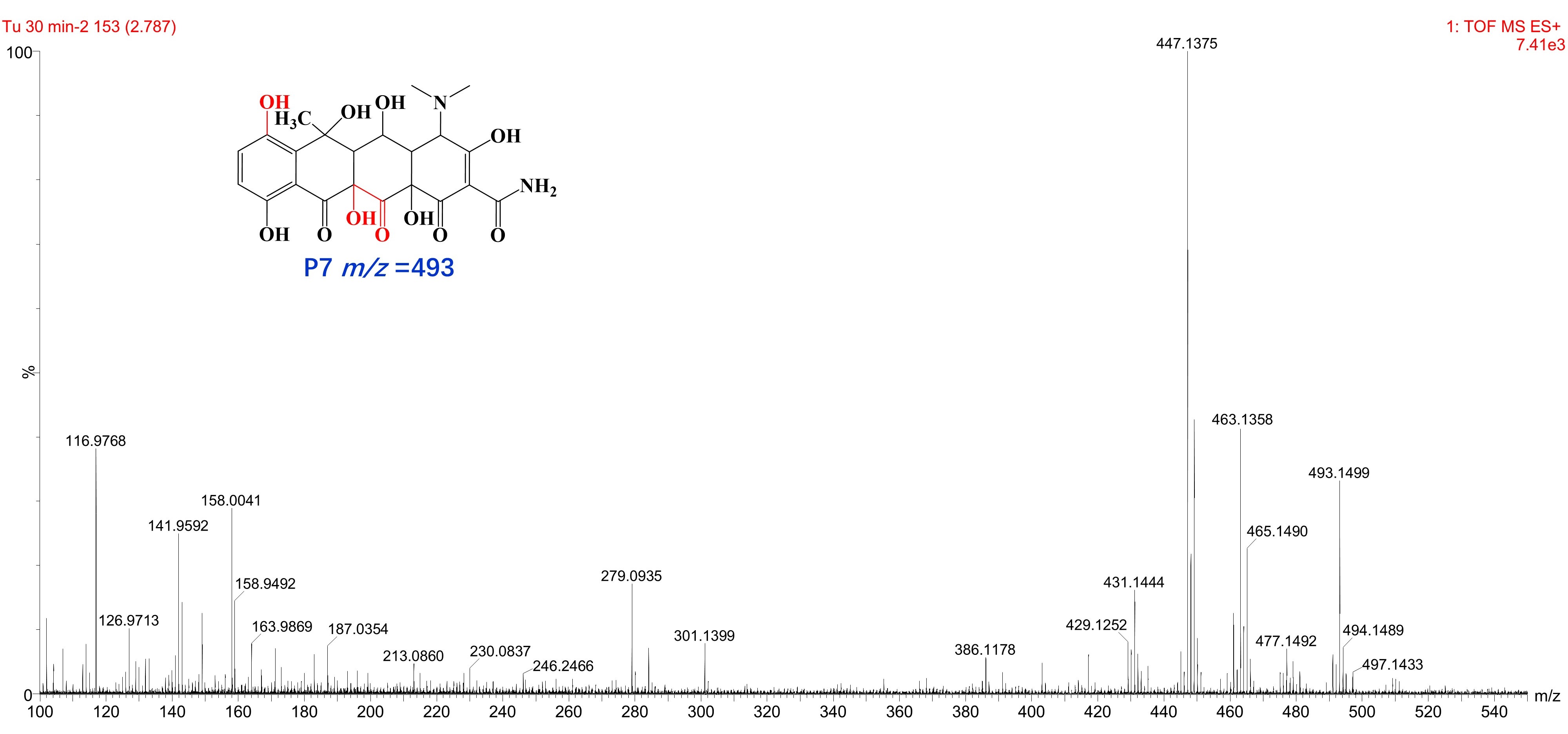

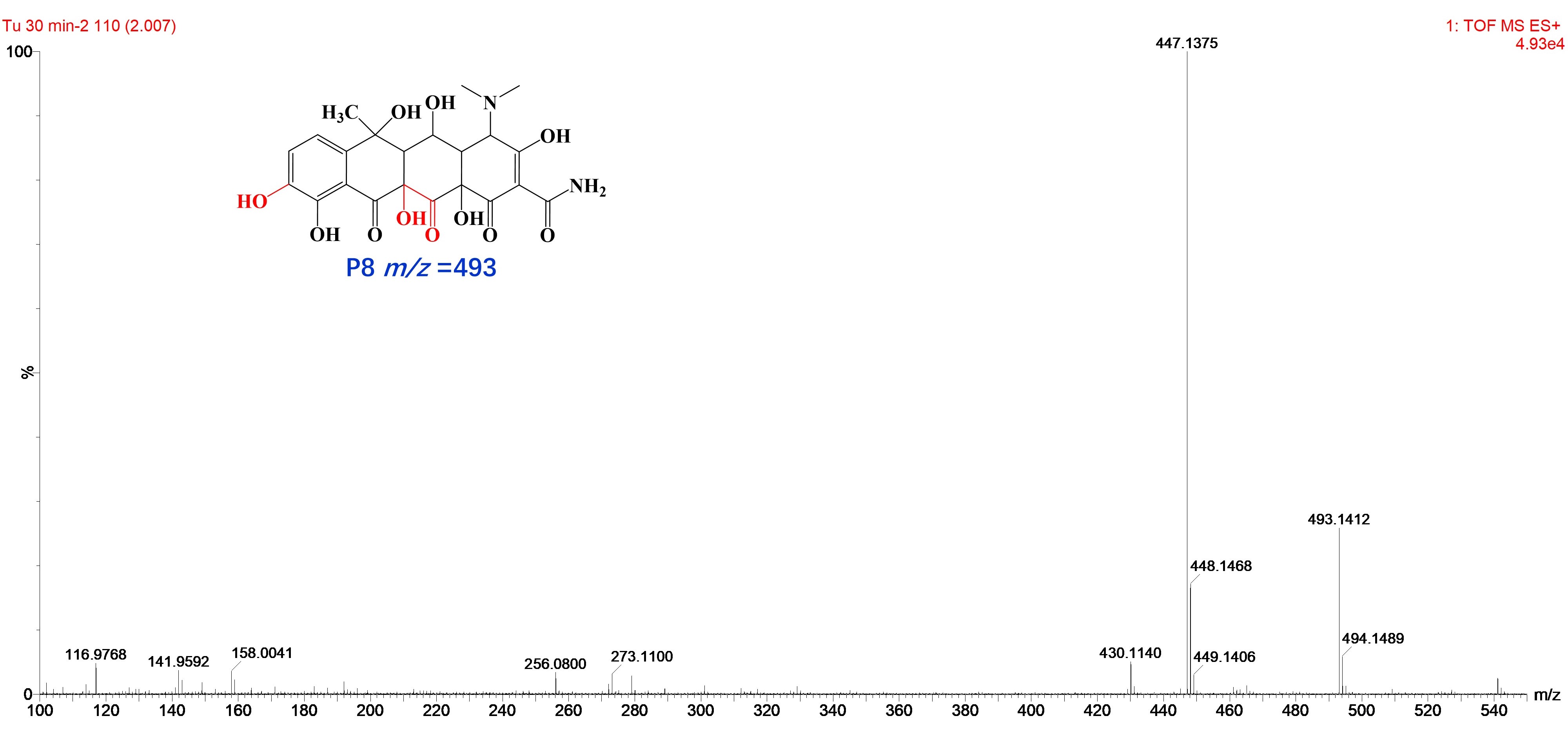

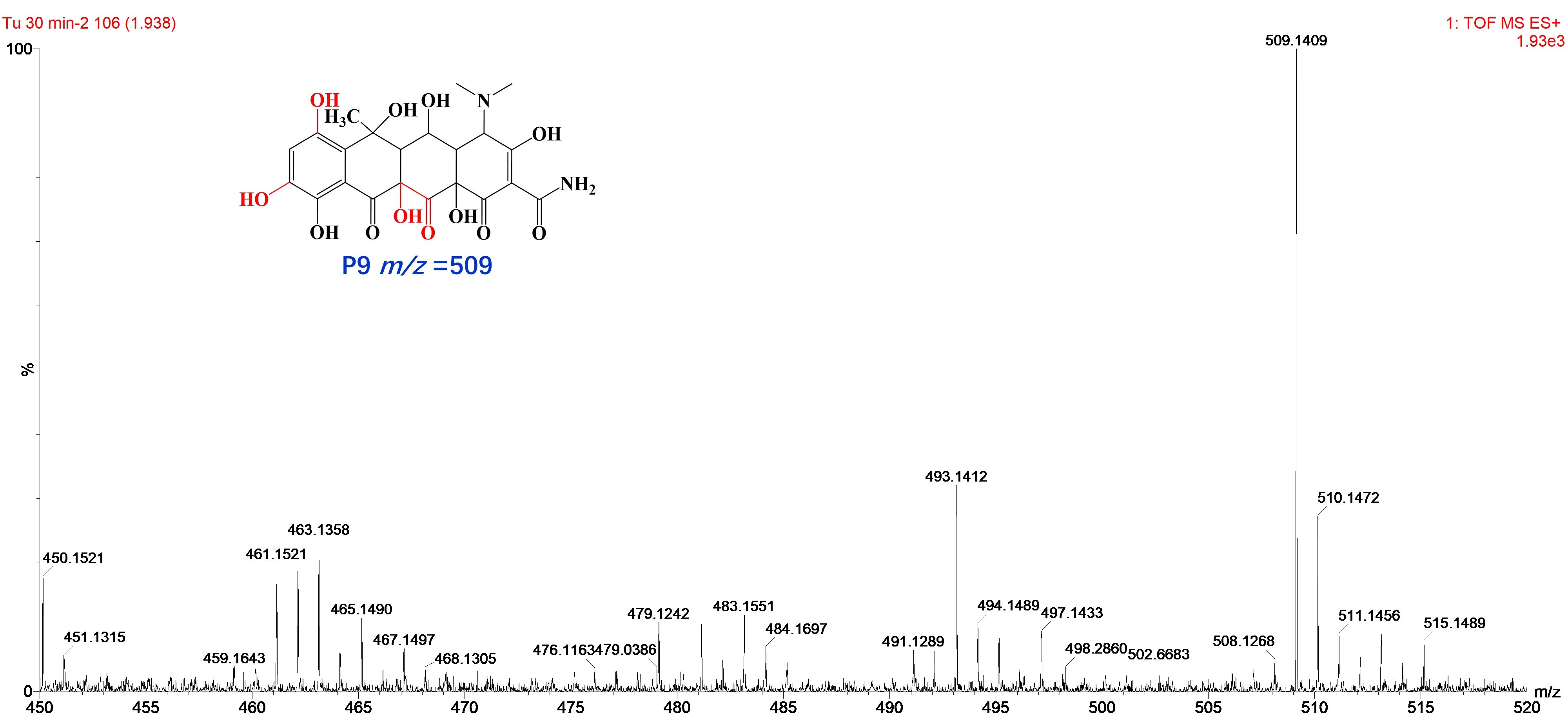

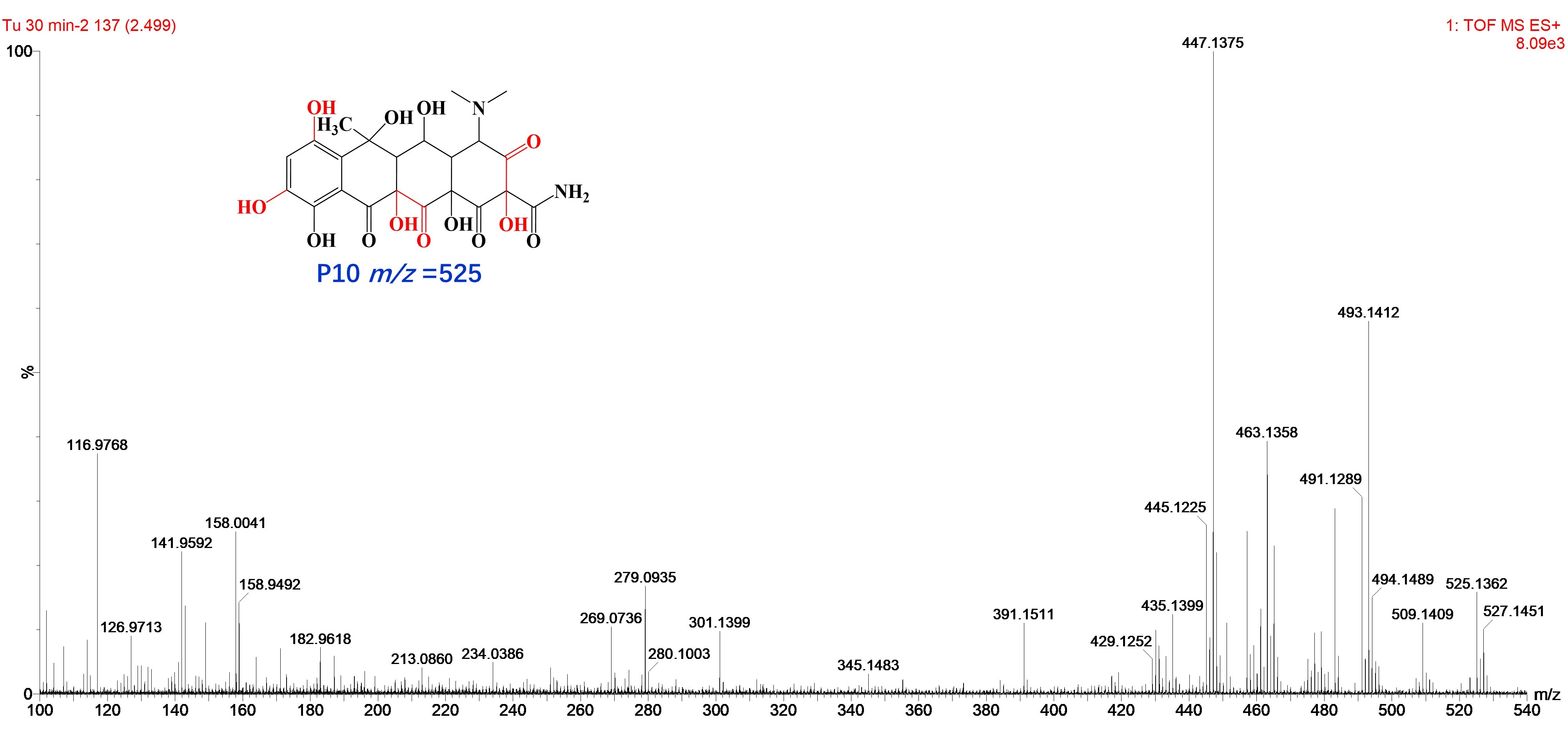

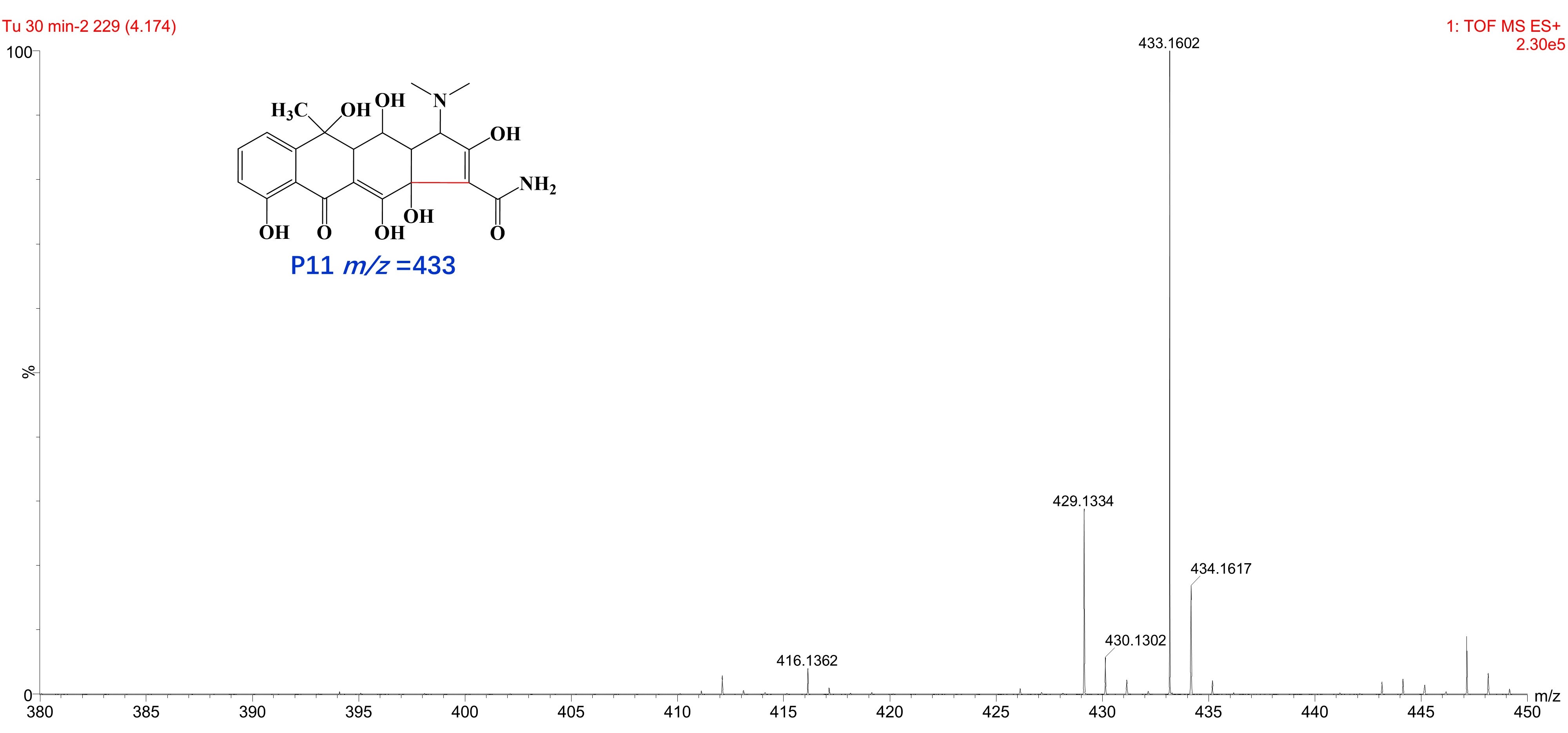

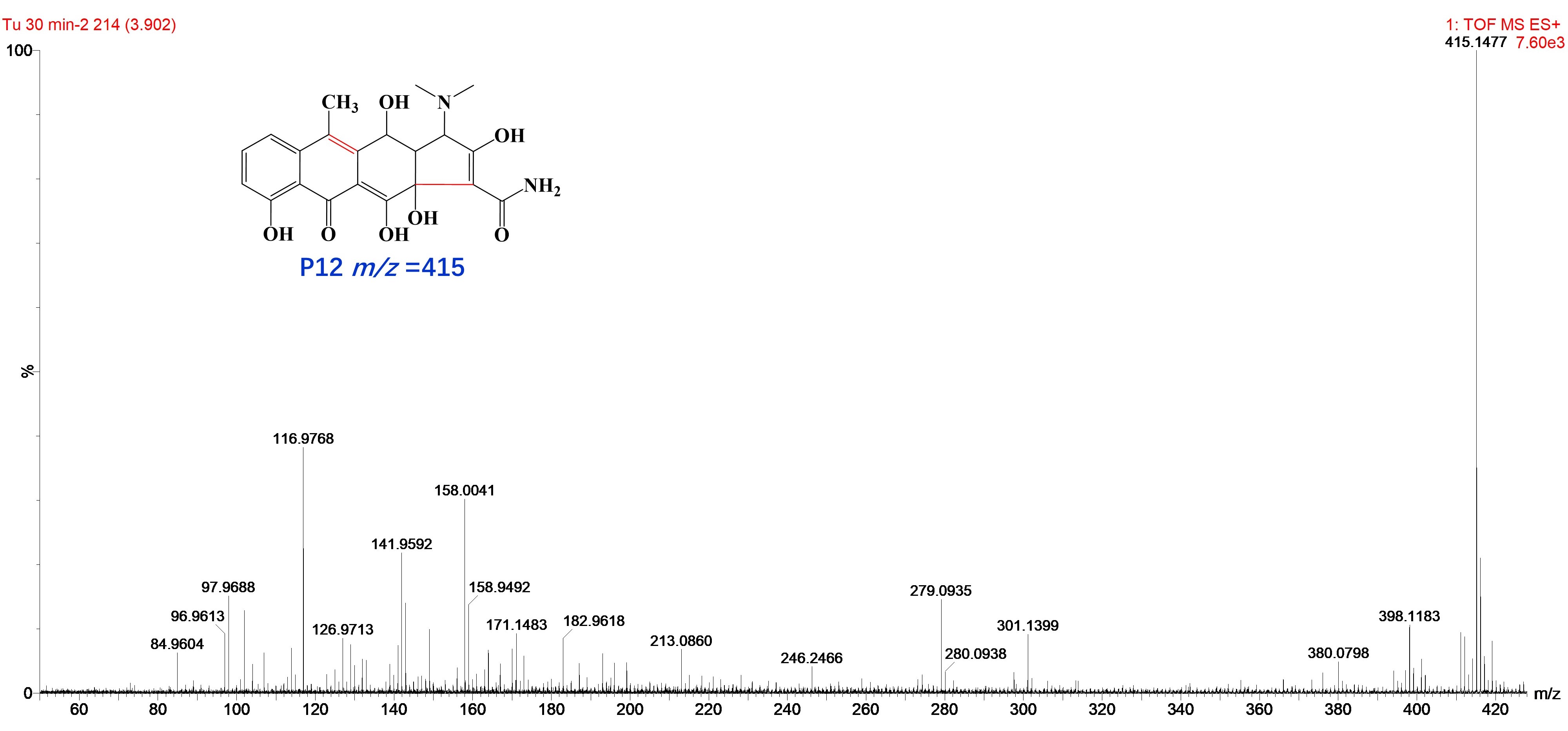

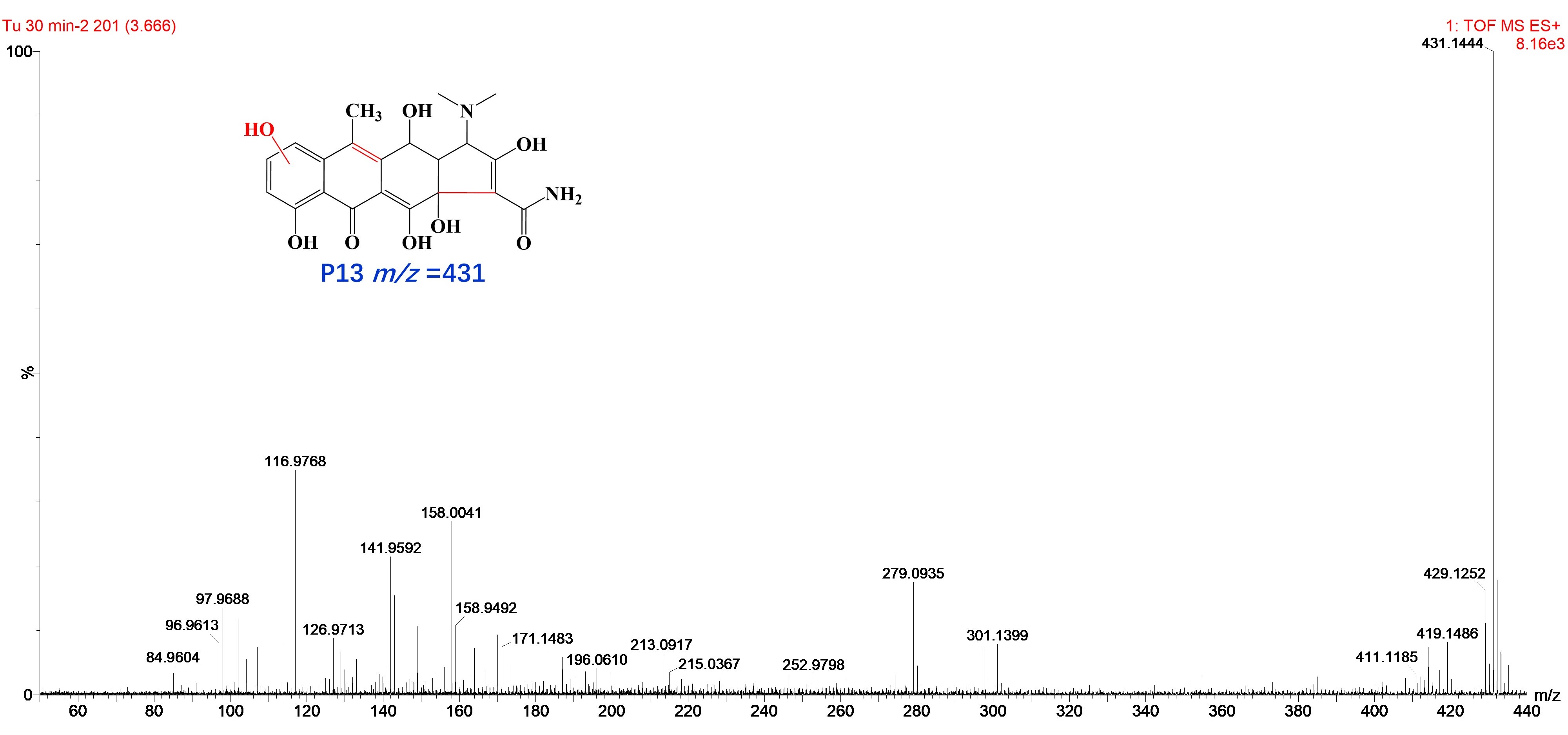

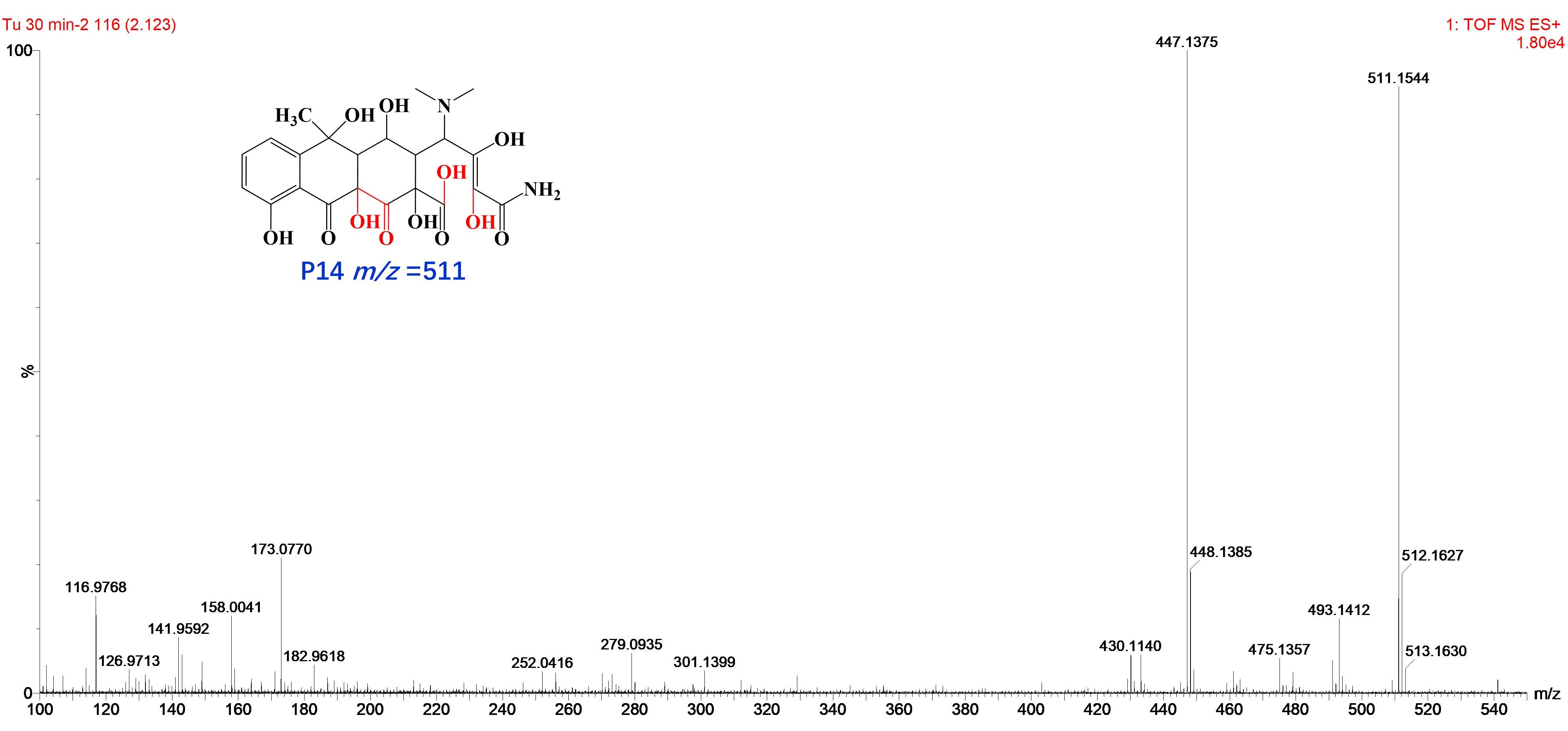

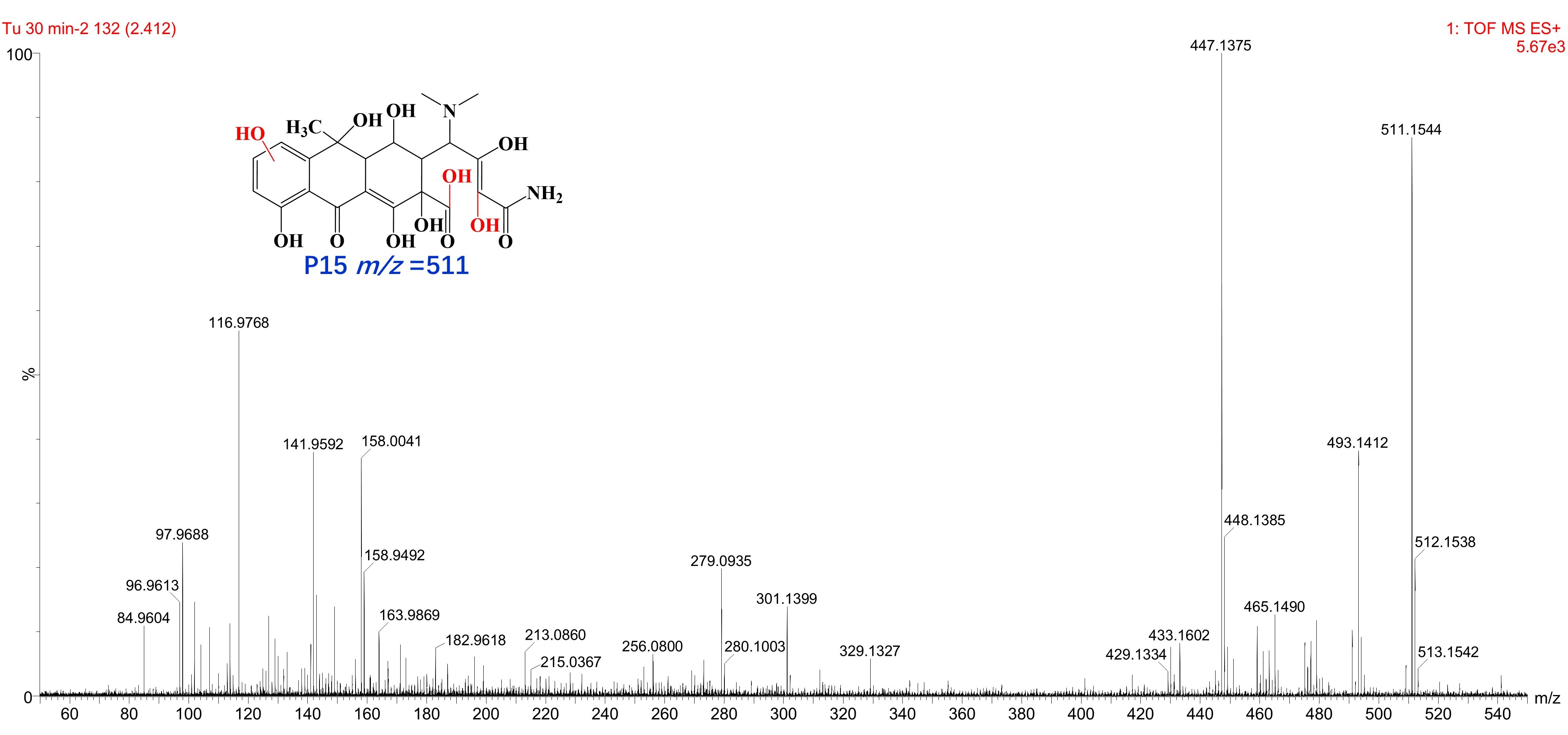

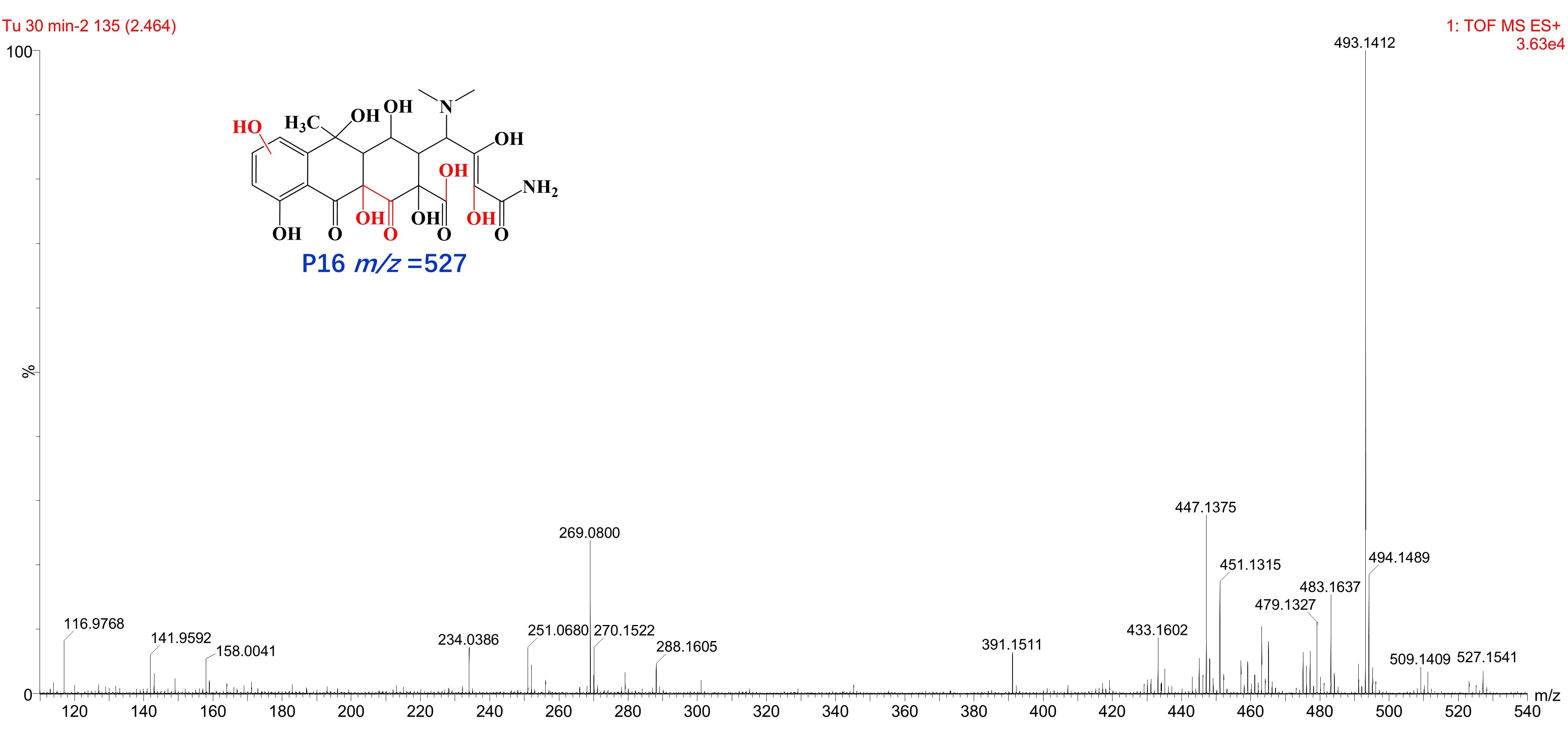

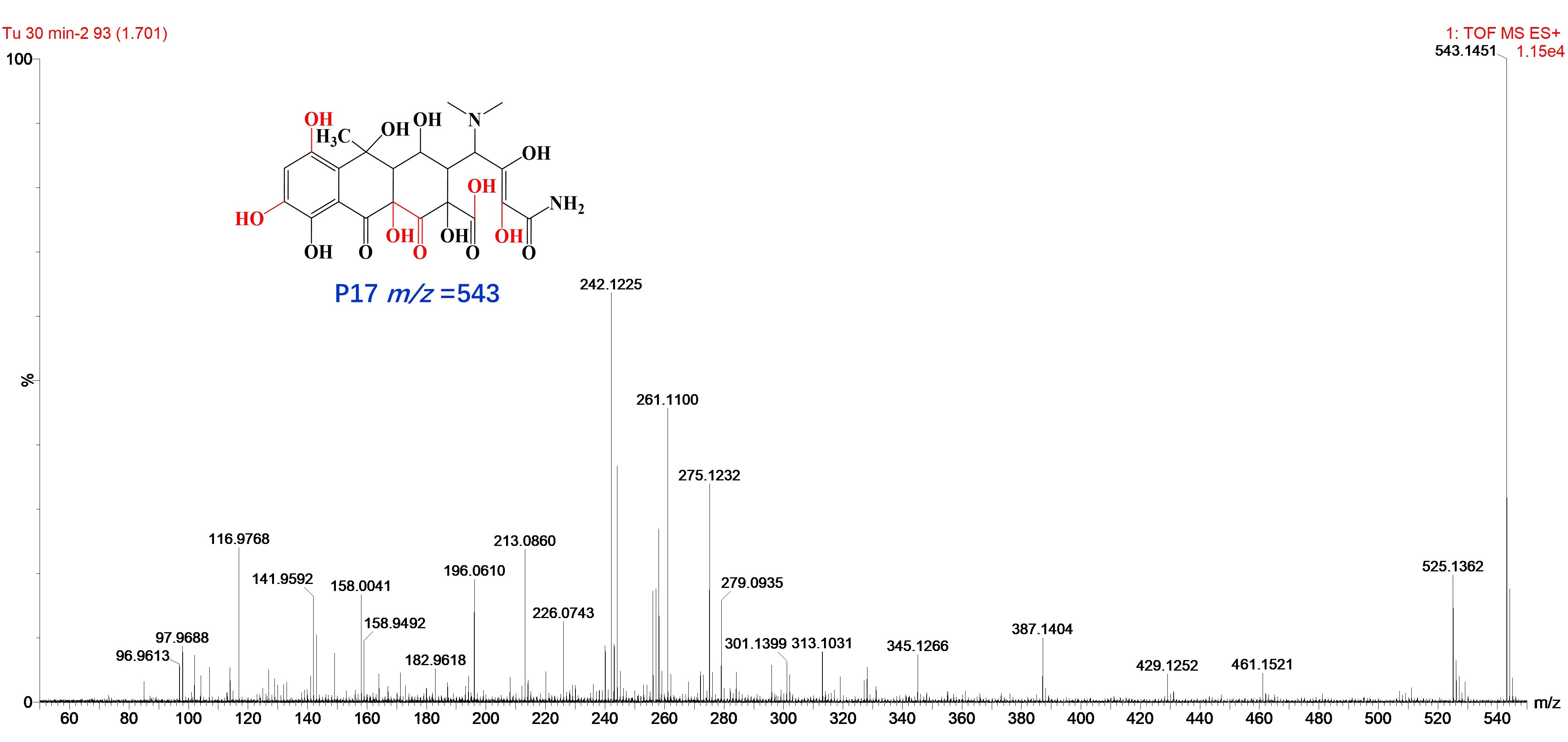

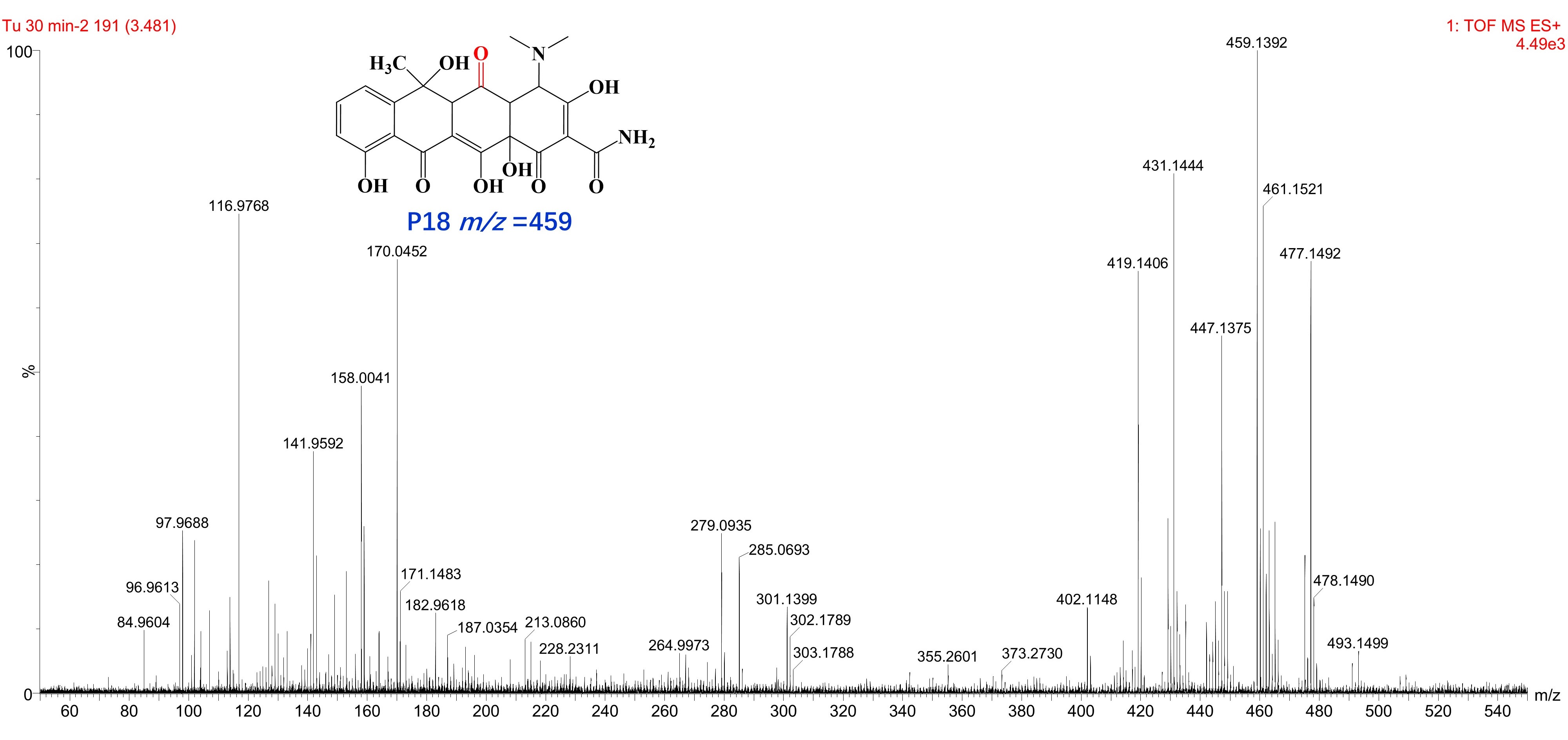

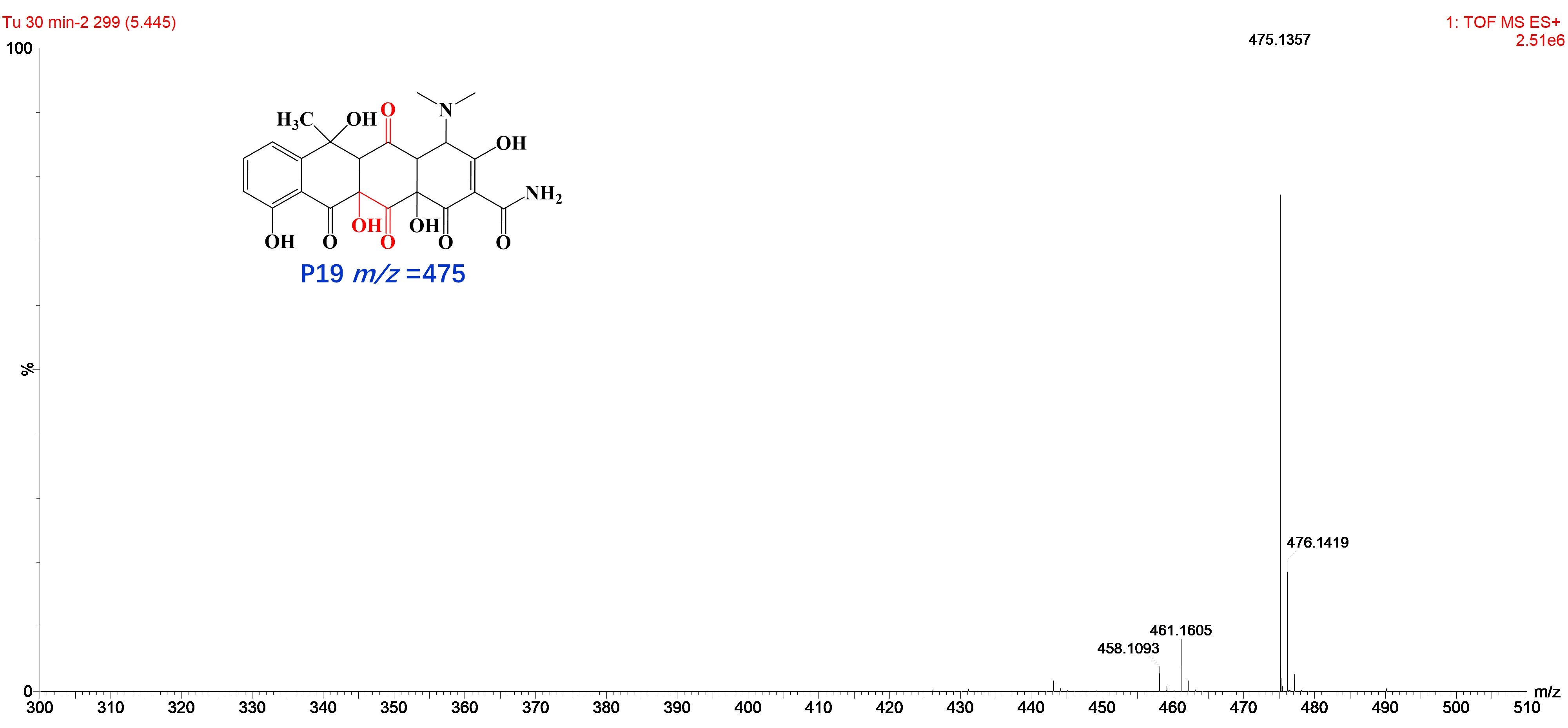

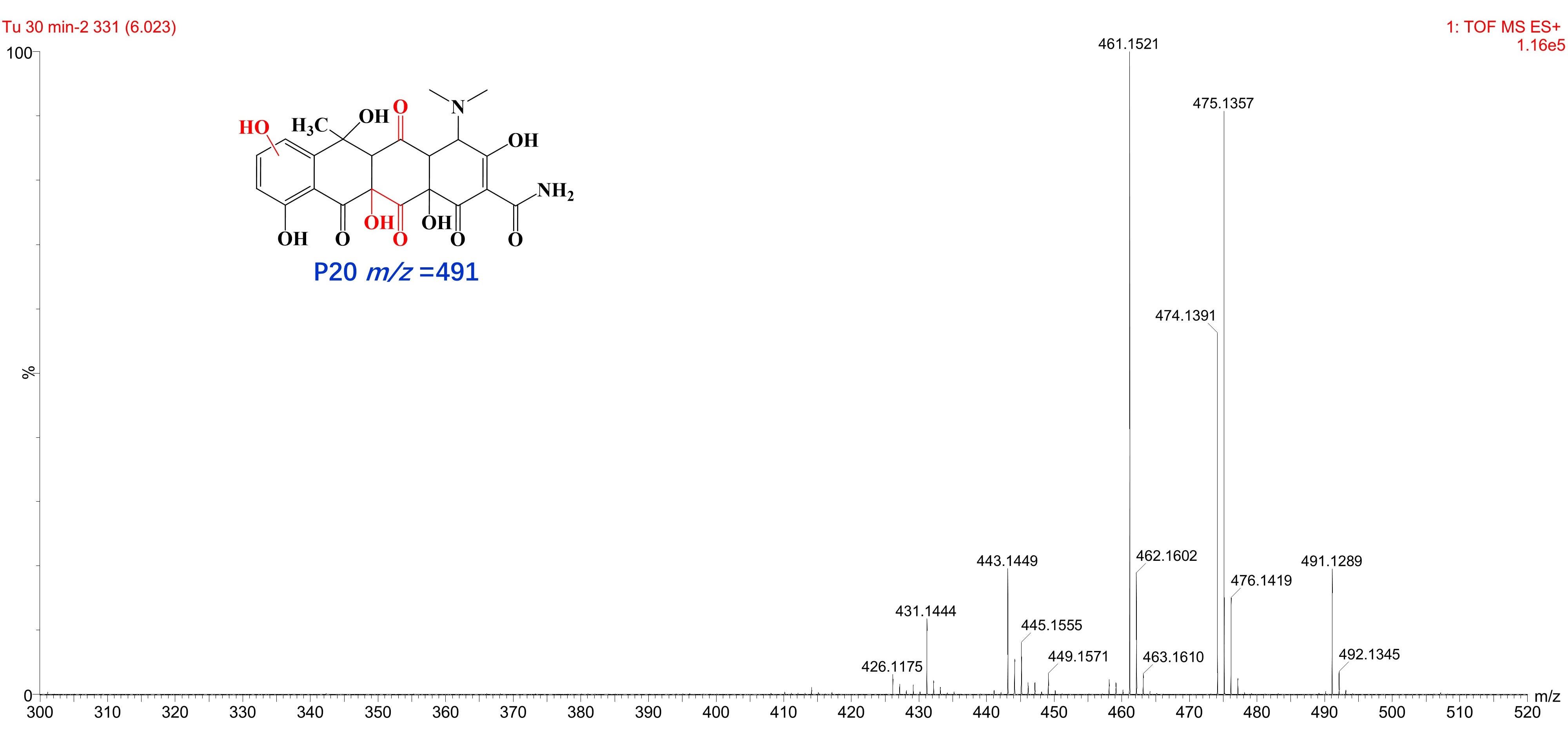


**Figure S5** The UPLC-Q-TOF MS spectra of OTC and its transformation products.


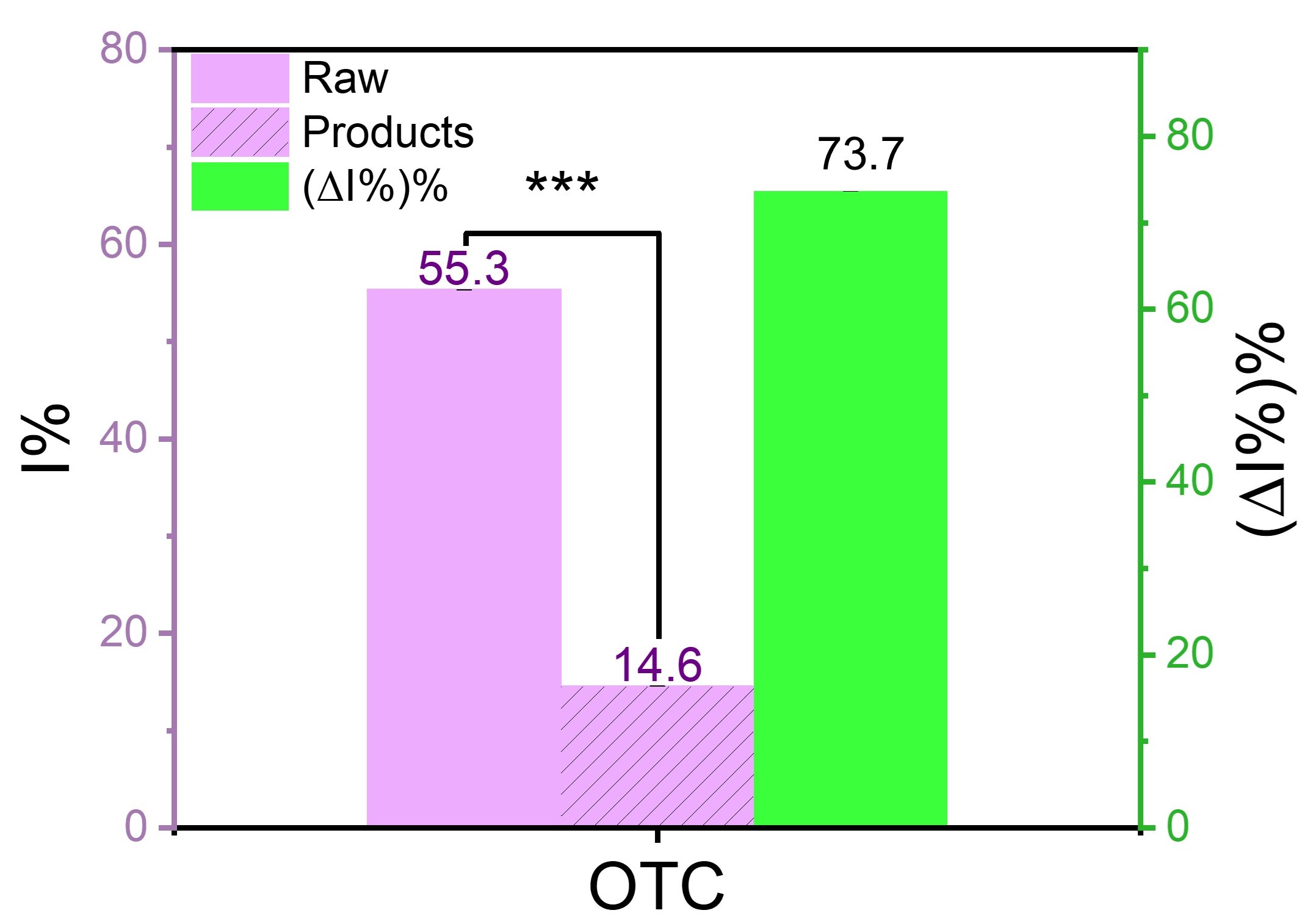


**Figure S6** I% and (ΔI%)% of luminous bacteria treated by OTC and its transformation products by C-TiO_2_/SiNPs/LED system. (n = 3 independent samples, data are presented as the mean ± S.D., one-way ANOVA, ****p* < 0.001).
